# Identification of Transcription Factors ZmMYB111 and ZmMYB148 Involved in Phenylpropanoid Metabolism

**DOI:** 10.3389/fpls.2016.00148

**Published:** 2016-02-15

**Authors:** Junjie Zhang, Shuangshuang Zhang, Hui Li, Hai Du, Huanhuan Huang, Yangping Li, Yufeng Hu, Hanmei Liu, Yinghong Liu, Guowu Yu, Yubi Huang

**Affiliations:** ^1^College of Life Science, Sichuan Agricultural UniversityChengdu, China; ^2^College of Agronomy, Sichuan Agricultural UniversityChengdu, China; ^3^College of Agronomy and Biotechnology, Southwest UniversityChongqing, China; ^4^Maize Research Institute, Sichuan Agricultural UniversityChengdu, China

**Keywords:** maize, MYB, transcription factor, phenylpropanoid metabolism, gene expression

## Abstract

Maize is the leading crop worldwide in terms of both planting area and total yields, but environmental stresses cause significant losses in productivity. Phenylpropanoid compounds play an important role in plant stress resistance; however, the mechanism of their synthesis is not fully understood, especially in regard to the expression and regulation of key genes. Phenylalanine ammonia-lyase (PAL) is the first key enzyme involved in phenylpropanoid metabolism, and it has a significant effect on the synthesis of important phenylpropanoid compounds. According to the results of sequence alignments and functional prediction, we selected two conserved R2R3-MYB transcription factors as candidate genes for the regulation of phenylpropanoid metabolism. The two candidate R2R3-MYB genes, which we named ZmMYB111 and ZmMYB148, were cloned, and then their structural characteristics and phylogenetic placement were predicted and analyzed. In addition, a series of evaluations were performed, including expression profiles, subcellular localization, transcription activation, protein–DNA interaction, and transient expression in maize endosperm. Our results indicated that both ZmMYB111 and ZmMYB148 are indeed R2R3-MYB transcription factors and that they may play a regulatory role in PAL gene expression.

## Introduction

Maize is the leading crop worldwide in terms of both planting area and total yield (reached about 0.87 billion tons in 2012) according to the report of FAOFTAT^1^, and its production continues to expand in both developed and developing countries (Wang et al., 2013). It is used not only as a staple food source, but also for animal feed, biofuel, and various industrial raw materials. However, environmental stresses, such as extremes in temperature, drought, salinity, heavy metals, and oxidative stress, cause significant losses in maize productivity. To cope with these types of stress, plants are equipped with various mechanisms that enable them to survive harsh environmental conditions (Bohnert et al., 1995), including the ability to synthesize special compounds, such as phenylpropanoids (Solecka, 1997; Ramakrishna and Ravishankar, 2011). Therefore, a better understanding of the mechanism or phenylpropanoid synthesis is critical to improving the stress tolerance of maize.

Phenylpropanoid metabolism gives rise to a diverse group of compounds, which are derived from the carbon skeleton of phenylalanine and are involved in plant defense, structural support, and survival (Vogt, 2010). The phenylpropanoid pathway begins with three reactions that are respectively catalyzed by phenylalanine ammonia-lyase (PAL; EC 4.3.1.5), Cinnamate 4-hydroxylase (C4H; EC 1.14.13.11), and 4-coumarate: CoA ligase (4CL; EC 6.2.1.12), leading to the synthesis of p-coumaroyl CoA (Fraser and Chapple, 2011), which is a common precursor for the production of many other important compounds. Lignin and flavonoid are the main phenylpropanoid compounds found. In maize, lignin is mainly distributed in the stem, root, and leaf tissues, and flavonoids are mainly located in seeds.

One important group of secondary metabolites that is derived from the phenylpropanoid pathway is the flavonoids, which is a group of polyphenolic secondary metabolites that includes the anthocyanins (red to purple pigments), flavonols (colorless to pale yellow pigments), flavonols (colorless pigments that become brown after oxidation), and proanthocyanidins (PAs) or condensed tannins (Petrussa et al., 2013). Flavonoids play a major role in plant responses to both biotic and abiotic stresses. For example, when abiotic stressors (such as phosphate, nitrogen, light, temperature, UV, or drought) cause the accumulation of reactive oxygen species (ROS), flavonoids are able to effectively scavenge ROS, thus preventing oxidative damage (Nakabayashi et al., 2014).

Another important group of phenylpropanoid-derived compounds is the lignins, which is a large group of aromatic polymers that are major components of plant cell walls and that serve a variety of functions related to both structural support and plant defense (Boerjan et al., 2003). One of the major functions of lignins is protecting cell wall polysaccharides from microbial degradation (Mansfield, 2009; Vanholme et al., 2010), and the synthesis of lignins can be induced by multiple types of abiotic stress, including mineral deficiency, drought, UV-B radiation, and low temperature, as well as by biotic stressors, such as infection by fungi, bacteria, or viruses (Moura et al., 2010).

The myeloblastosis (MYB) proteins comprise one of the largest and most functionally diverse families of transcription factors in plants and are characterized by a highly conserved DNA-binding domain, the MYB domain, which is approximately 52 amino acid residues in length and is capable of forming a helix-turn-helix fold with three regularly spaced tryptophan residues (Kanei-Ishii et al., 1990; Katiyar et al., 2012). The majority of plant MYB proteins belong to the R2R3-MYB subfamily (Dubos et al., 2010; Cai et al., 2012; Katiyar et al., 2012), and the R2R3-MYB transcription factors play central roles in controlling plant-specific processes, including primary and secondary metabolism, cell fate and identity, development, and response to both abiotic and biotic stresses (Dubos et al., 2010; Katiyar et al., 2012).

In previous studies, we performed a genome-wide survey of the R2R3-MYB gene subfamily in maize, in which 158 putative R2R3-MYBs were identified, and a comprehensive analysis that included phylogenetics, expression patterns, and determination of the proteins’ structural and functional characteristics (Du et al., 2012). Our results indicated that protein functions were conserved between maize MYB genes and their putative orthologs, based on homology, co-expression, and collinearity analyses (Du et al., 2012). Accordingly, we selected two conserved R2R3-MYB transcription factors as candidate genes for the regulation of phenylpropanoid metabolism. The two candidate R2R3-MYB genes, which we named ZmMYB111 and ZmMYB148, were cloned from the maize inbred line B73, and their regulatory mechanisms were determined.

## Materials and Methods

### Plant Materials

Seeds for the B73 maize inbred line were provided by the College of Agronomy, Sichuan Agricultural University and were grown at the school farm in the summer of 2013, according to the local standards for maize production. When silks emerged, strict self-pollinations were performed every morning, and 25 days after pollination (DAP), roots, leaves, upper and lower stems, pith, and both male and female flowers were sampled for RNA isolation. All samples were immediately frozen in liquid nitrogen and stored at -70°C until used. Fresh endosperms were harvested at 9 DAP for use as receptors for transient expression.

### RNA Extraction, Reverse Transcription, and DNA Extraction

Total RNAs were extracted from the frozen samples using Trizol reagent (Invitrogen, China), and treated with gDNA Eraser (Takara, Dalian, China) to remove genomic DNA contamination. One μg of total RNA from each sample was used to produce cDNA for cloning both ZmMYB111 and ZmMYB148 and for measuring gene transcription levels via reverse transcription PCR (RT-PCR; PrimeScript^TM^ RT Reagent Kit; Takara, China). Genomic DNA (gDNA) was isolated from young leaves using the cetyltrimethylammonium bromide (CTAB) method (Fulton et al., 1995).

### Gene Cloning of ZmMYB111 and ZmMYB148 and Bioinformatics Analysis

The cDNAs of ZmMYB111 (GRMZM2G104551) and ZmMYB148 (GRMZM2G097636) were amplified from cDNA templates from the upper stem samples using the following primer pairs: 5′-GAATTCATGGGTCGGCAGCCGT-3′ (forward) and 5′-GAGCTCCTAGAACTTTGCTCCGTTTG-3′ (reverse), and 5′-GAATTCATGGGGAAGGGCC-3′ (forward) and 5′-GAGCTCTCACAACCCCATCTG-3′ (reverse). The primers were based on the gene sequences from NCBI^2^, designed using Primer 5 software, and synthesized by Invitrogen (Shanghai). The PCR amplification products were inserted into the pMD19-T cloning vector (Takara, Dalian, China) and sequenced by Majorbio^3^. Secondary structure, tertiary structure, typical domain of R2R3 MYB protein, motif, and phylogenetic tree analyses of the ZmMYB111 and ZmMYB148 proteins were predicted using online bioinformatics analysis and other software.

### Cloning Promoters of the PAL and 4CL Genes

Promoters (∼2000 bp) of the PAL (GRMZM2G029048) and 4-coumaric acid COA ligase (4CL, GRMZM2G075333) were amplified from maize gDNA using PCR with the following primer pairs for PAL and 4CL, respectively: 5′-GAATTCAAACCATGCGTCAGTTGAC-3′ (forward) and 5′-ACGCGTTGCGGTTGCGACAC-3′ (reverse), and 5′-GAATTCGACGAGTGATCAAAGGTT-3′ (forward) and 5′-ACGCGTCTCAGACCTTTGCTC-3′ (reverse). The PCR products were inserted into the pMD19-T cloning vector (Takara, Dalian, China) and sequenced by Majorbio^4^.

### Transcription Levels

Quantitative real-time PCR (qRT-PCR) analysis was performed using SYBR Premix Ex TaqTM II (Tli RNaseH Plus; Clontech) according to the manufacturer’s protocol on a CFX96TM Real-Time PCR system (Bio-Rad, Hercules, CA, USA). The qRT-PCR reaction mixtures contained 0.5 μL (0.4 μM) each primer, 1.0 μL cDNA, 8 μL ddH_2_O, and 10 μL SYBR Green II in a total volume of 20 μL.

Primers for the three target genes (*ZmMYB111*, *ZmMYB148*, and *PAL*) were designed to amplify fragments of approximately 200 bp, and the maize actin gene (*Actin*) was used as a reference (**Table 1**). The qRT-PCRs were carried out in biological triplicates, and standard curves were verified with CFX Manager 3.1(Bio-Rad, USA) using a linear regression analysis of the threshold cycle CT value for each of 8 total DNA standard dilutions in each sample. The specificity of PCR primers was determined using melting curve analysis of the amplified products. The maize *actin* gene was used as the reference gene to normalize the expression of the target genes, which was calculated using the relative quantization method (2^-Δ*Ct*^).

**Table 1:** Real time PCR primer sequences for the three targeted genes and *Actin*.

Gene name	JGI number	Primer sequence (5′–3′)
*ZmMYB111*	GRMZM2G104551	F: GAACGACGACGCCGTGATAAG
		R: TCTTCCAAAAGCCACTTCACCAG
*ZmMYB148*	GRMZM2G097636	F: GCGGAGGGAAAGGAGTGGTG
		R: GCCCATTGGGTACTGGTAGTCC
*PAL*	GRMZM2G029048	F: CGTCAACGACAACCCGCTCA
		R: GAGGCCGTTGTTGTAGTAGTCGTTC
*Actin*	GRMZM2G320797	F: TCACTACGACTGCCGAGCGAG
		R: GAGCCACCACTGAGGACAACATTAC


### Subcellular Localization

The GFP-ZmMYB111 and GFP-ZmMYB148 vectors were constructed to visualize subcellular localization. The full-length cDNAs of ZmMYB111 and ZmMYB148 were amplified by PCR using the following primers: 5′-CGGGATCCATGGGTCGGCAGCCGT-3′ (forward) and 5′-GCTCTAGACTAGAACTTTGCTCCGT-3′ (reverse), and 5′-CGGGATCCATGGGGAAGGGCC-3′ (forward) and 5′-GCTCTAGATCACAACCCCATCTG-3′ (reverse). (The underlined sections in the forward and reverse primer sequences indicate *Bam*HI and *Xba*I sites, respectively.) The PCR products were inserted into the pMD19-T cloning vector, and after digestion with *Bam*HI and *Xba*I, the resulting fragments were ligated into the pCAMBIA2300-35S-eGFP vector, which contained a GFP protein driven by the CaMV 35S promoter (plasmid map in Supplementary Datasheet), amplified using PCR, sequenced, and named GFP-ZmMYB111 and GFP-ZmMYB148 after identification.

Onion epidermal cells were then bombarded with the GFP-Zma111 and GFP-Zma148 vectors, respectively, using a helium biolistic gun transformation system (Bio-Rad, USA), as described previously (Hu et al., 2012). The transformed cells were incubated on 1/2 MS medium for 24–48 h at 28°C. The subcellular localization of GFP fusion proteins was visualized using a BX61 fluorescence microscope with a wavelength of 488 nm (Olympus, Japan). Empty p2300-35S-eGFP vector was used as a control.

### Transcription Activation

The pGBDKT7-ZmMYB111 and pGBDKT7-ZmMYB148 vectors were constructed for transcription activation analysis. The full-length cDNAs of ZmMYB111 and ZmMYB148 were amplified using PCR with the following primers: 5′-CGGAATTCATGGGTCGGCAGCCGT-3′ (forward) and 5′-CGGGATCCCTAGAACTTTGCTCCGT-3′ (reverse), and 5′-CGGAATTCATGGGGAAGGGCC-3′ (forward) and 5′-CGGGATCCTCACAACCCCATCTG-3′ (reverse). (The underlined sections in the forward and reverse primer sequences indicate *Eco*RI and *Bam*HI *s*ites, respectively.) The PCR products were inserted into the pMD19-T cloning vector, and after digestion with *Eco*RI and *Bam*HI, the resulting fragments were ligated into the pGBKT7 DNA-binding domain vector (Clontech, USA), amplified using PCR, sequenced, and named PGBDKT7-ZmMYB111 and PGBDKT7-ZmMYB148 after identification.

Transcription activation analysis was performed in order to assess the presence of a protein activation domain (Fujita et al., 2004). For this analysis, both pGADT7-ZmaMYB111 and pGADT7-ZmaMYB148 vectors were transformed into AH109 yeast cells using the lithium acetate-mediated method, respectively (Gietz and Woods, 2002). The transformants were screened on SD/-Trp medium, and positive clones were confirmed using PCR. The colonies were then screened on SD/-Trp-His-Leu medium with X-α-gal. In addition, the yeasts were further cultivated at 28°C for 3–5 days to test transcription activation. The pGBDKT7-Lam and pGBDKT7-53 vectors and AH109 (no vector) were used as controls.

### Protein–DNA Interaction

The yeast one-hybrid system is widely recognized as a valuable and straightforward technique for studying interactions between transcription factors and DNA. Accordingly, pGADT7-ZmaMYB111 and pGADT7-ZmaMYB148 vectors were constructed for use in a yeast one-hybrid assay. The cDNAs of ZmMYB111 and ZmMYB148 were amplified using PCR with the following primers: 5′-CGGAATTCATGGGTCGGCAGCCGT-3′ (forward) and 5′-CGAGCTCCTAGAACTTTGCTCCGT-3′ (reverse), and 5′-CGGAATTCATGGGGAAGGGCC-3′ (forward) and 5′-CGAGCTCTCACAACCCCATCTG-3′ (reverse). (The underlined sections in the forward and reverse primer sequences indicate *Eco*RI and *Sac*I sites, respectively.) The PCR products were inserted into the pMD19-T cloning vector, and after digestion with *Eco*RI and *Sac*I, the resulting fragments were ligated into the multiple cloning site (MCS) of the pGADT7-Rec2 vector (Clontech, USA), amplified using PCR, sequenced, and named pGADT7-ZmaMYB111 and pGADT7-ZmaMYB148 after identification.

In addition, the promoters for PAL and 4CL were ligated into the *Eco*RI and *Mlu*I restriction sites, respectively, of the pHis2 plasmid (Clontech, USA), amplified using PCR, sequenced, and named pHis2-pPAL and pHis2-p4CL after identification. The related recombinant expression vectors were co-transformed into the Y187 yeast strain. Then, growth of the transformed yeast were compared on SD/-Trp/-Leu medium and SD/-Leu-Ura-His+ with 10 mM 3-AT (3-amino-1,2,4-triazole) medium to test the expression of the His reporter gene. Empty pHis2 was used as a negative control.

### Transient Expression Assays in Maize Endosperm

The pBI221-ZmaMYB111 and pBI221-ZmaMYB148 vectors were constructed for transient expression analysis in maize endosperm. The cDNAs of ZmMYB111 and ZmMYB148 were amplified using PCR with the following primers: 5′-CCAAGCTTATGGGTCGGCAGCCGT-3′ (forward) and 5′-CGGGATCCCTAGAACTTTGCTCCGT-3′ (reverse), and 5′-CCAAGCTTATGGGGAAGGGCC-3′ (forward) and 5′-CGGGATCCTCACAACCCCATCTG-3′ (reverse). (The underlined sections in the forward and reverse primer sequences indicate *Hind*III and *Bam*HI sites, respectively.) The PCR products were inserted into the pMD19-T cloning vector, and after digestion with *Hind*III and *Bam*HI, the resulting fragments were ligated into the MCS of the pBI221 vector, in order to drive the LUC reporter gene (which replaced the GUS reporter gene), amplified using PCR, sequenced, and named pBI221-ZmaMYB111 and pBI221-ZmaMYB148 after identification.

Meanwhile, the promoters of PAL and 4CL were ligated into the *Xba*I and *Sac*I restriction sites, respectively, of the pBI221-ADH plasmid, which contained the first intron of the AdhI gene in order to enhance promoter activity without altering promoter specificity, amplified with PCR, sequenced, and named pBI221-pPAL-ADH after identification. These were also used for transient expression.

Endosperm was isolated from immature (9 DAP) maize kernels that had been surface-sterilized with 75% ethanol and was placed in MS medium (Murashige and Skoog salts containing 1% agar and 10% sucrose) for 4 h prior to bombardment. A Biolistic PDS-1000/He Particle Delivery System (Bio-Rad) was used to deliver gold particles coated with DNA. Each independent experiment consisted of four replicates, and was repeated 2–3 times with similar results. The bombarded endosperms were then cultivated for 36 h in order to analyze expression of the LUC reporter gene. Empty pBI221 vector, which contained the GUS gene driven by a maize ubiquitin promoter, was used as a control in order to correct for transfection efficiency. The fluorogenic assay for GUS activity was performed as previously described (Lu et al., 1998), and LUC activity was determined using a luciferase assay system (Promega, Madison, WI, USA). The fluorescence and luminescence were determined using a Luminoskan^TM^ Ascent (Thermo, Rockford, IL, USA), as described in our previous report (Hu et al., 2012).

## Results

### Cloning and Bioinformatics Analysis

**FIGURE 1:**
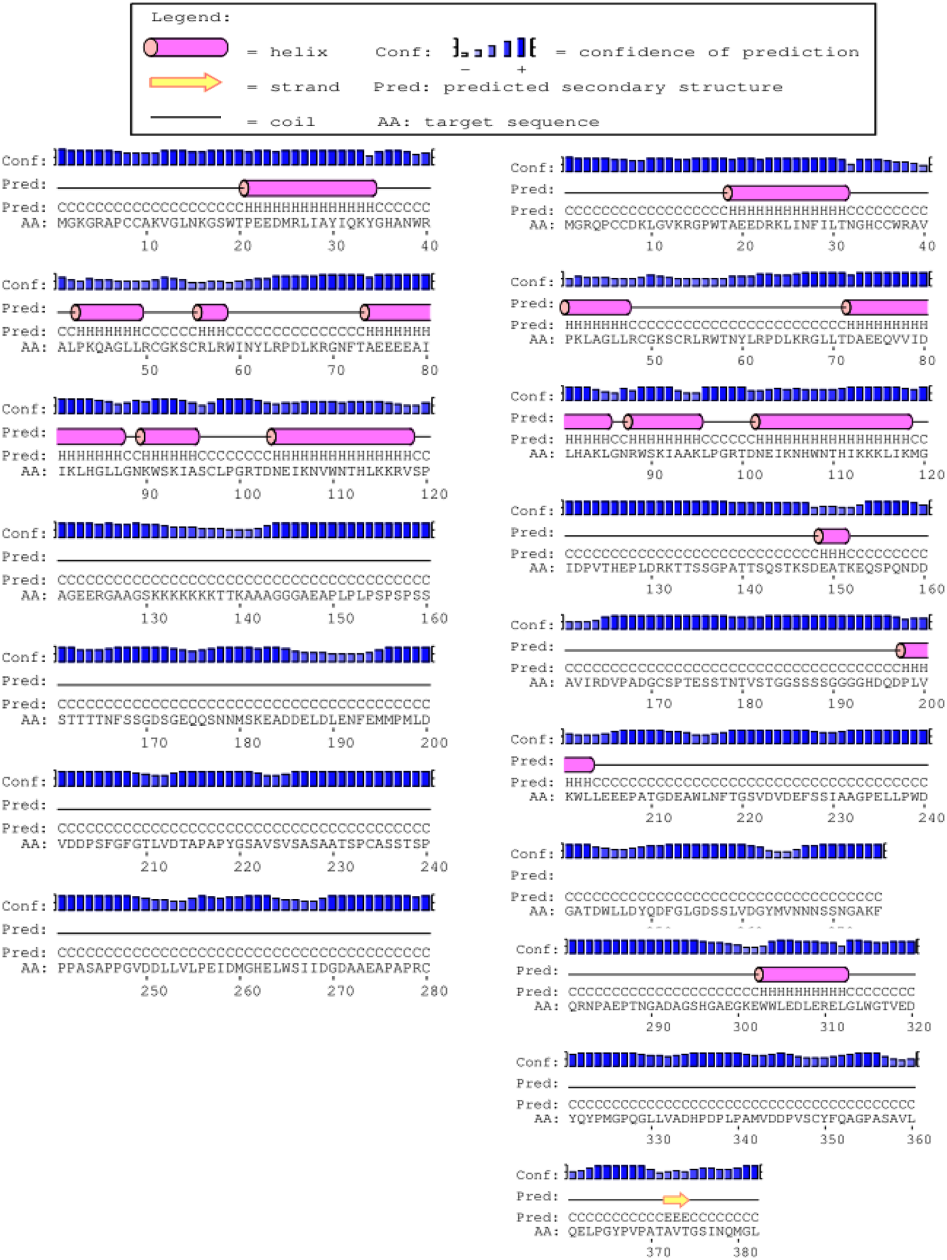
**Secondary structures of the amino acid sequences of *ZmMYB111* (left) and *ZmMYB148* (right), as predicted by PSIPRED analysis (http://bioinf.cs.ucl.ac.uk/psipred/)**.

**FIGURE 2:**
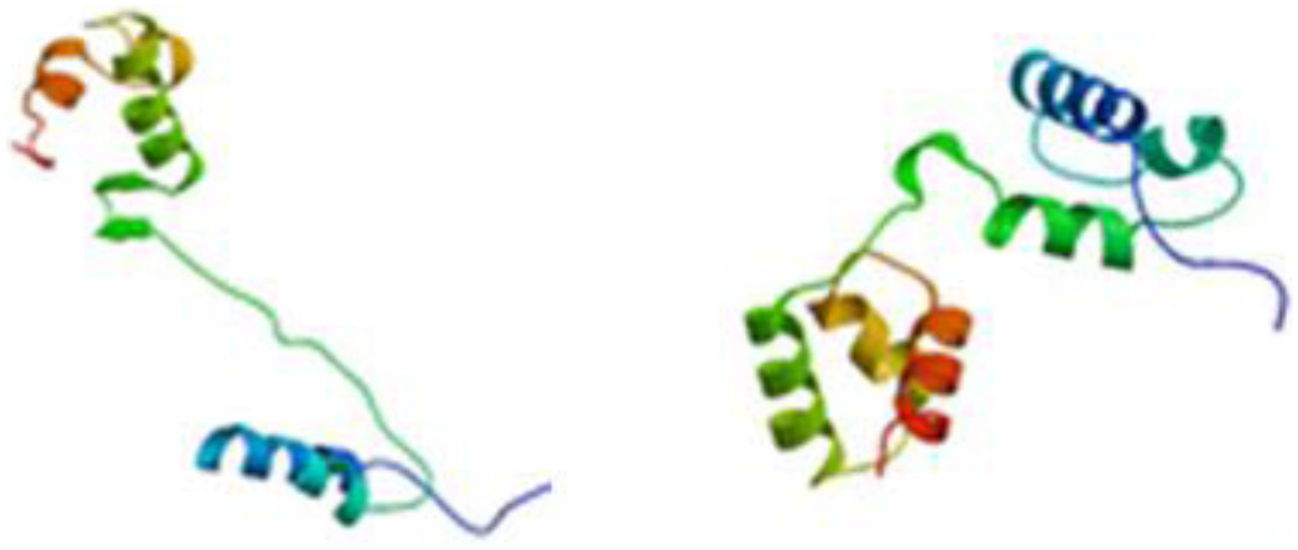
**Tertiary structure of ZmMYB111 (left) and ZmMYB148 (right), as predicted by SWISS-MODEL analysis (http://swissmodel.expasy.org/)**.

**FIGURE 3:**
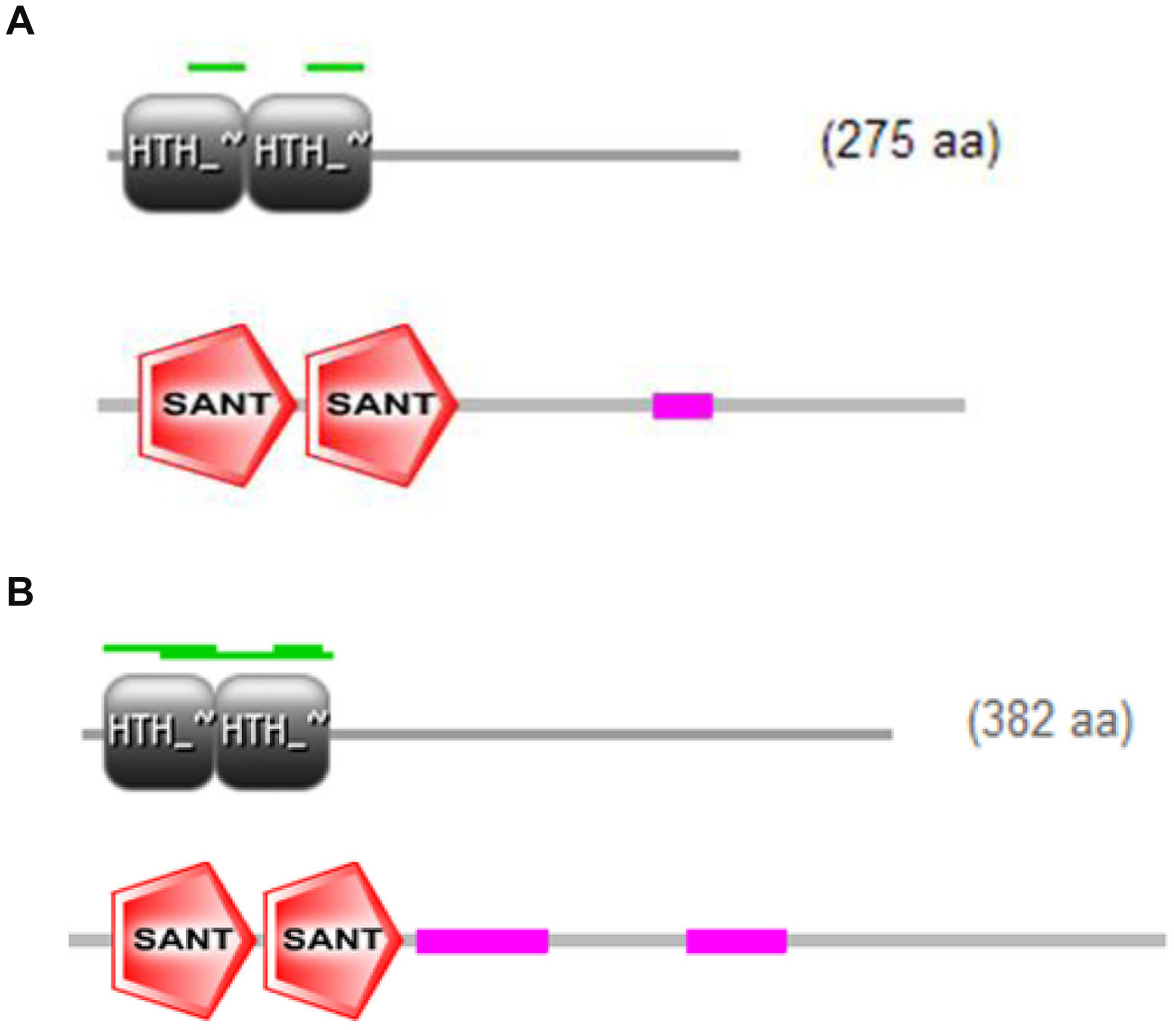
**Intra-domain features of ZmMYB111 **(A)** and ZmMYB148 **(B)**, as predicted by Expasy-Prosite analysis (http://prosite.expasy.org/)**.

The full-length cDNA sequences of ZmMYB111 and ZmMYB148 were amplified by RT-PCR, and the PCR products were analyzed using agarose gel electrophoresis. Sequencing revealed that the cDNA sequences of ZmMYB111 and ZmMYB148 were consistent with the GenBank report. The ZmMYB111 gene contains 828 bases and encodes a protein with 275 amino acids, an isoelectric point of 5.44, and a putative molecular weight of 30.05 kDa. In contrast, the ZmMYB148 gene contains 1149 bases and encodes a protein with 382 amino acids, an isoelectric point of 5.01, and a putative molecular weight of 40.65 kDa. Analysis using the PSIPRED software^5^ revealed that the n-termini of both proteins mainly consists of an α-helix (**Figure 1**), including three α-helix repeats that form a helix-turn-helix (HTH) motif as the DNA-binding domain, according to analysis using the SWISS-MODEL software^6^ (**Figure 2**), and the HTH motif is the MYB transcription factor typical characteristics. In addition, through domain analysis using Expasy-Prosite^7^, we found that the ZmMYB111 and ZmMYB148 proteins included the conserved domain typical of R2R3-MYB transcription factors (**Figure 3**). Together, the above results confirmed that both ZmMYB111 and ZmMYB148 proteins belong to the MYB transcription factor family.

**FIGURE 4:**
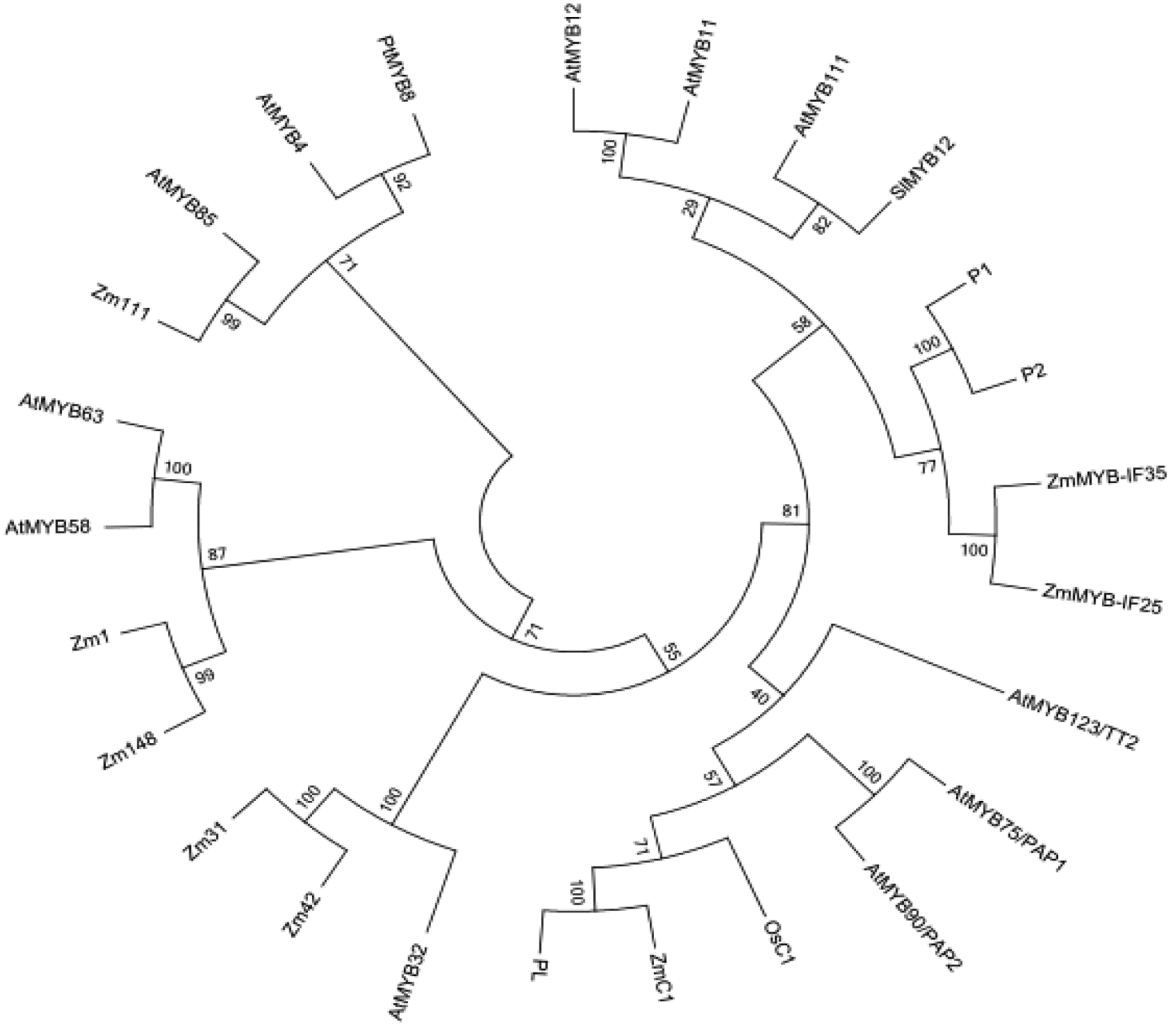
**Phylogenetic tree of ZmMYB111 and ZmMYB148 with other MYB transcription factors, based on amino acid sequences and built using MAFFT (http://mafft.cbrc.jp/alignment/server/index.html) and MEGA5.0 software.** The tree was generated using the neighbor-joining method with bootstrap support by 1000 replicates.

**FIGURE 5:**
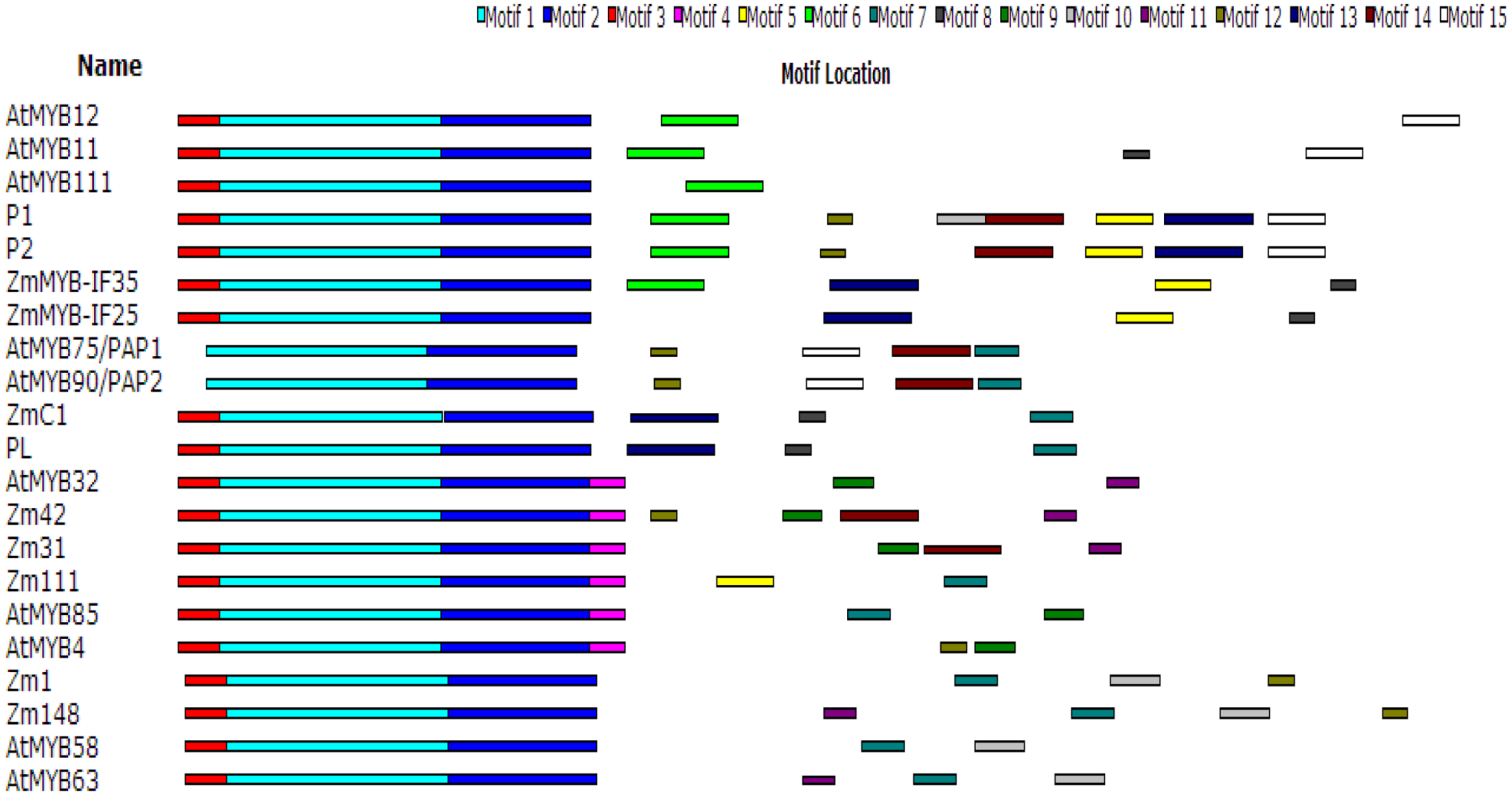
**Conservative motif analysis of ZmMYB111and ZmMYB148 using MEME software (http://meme.nbcr.net/meme/)**.

A phylogenetic (NJ) tree of the amino acid sequences of ZmMYB111, ZmMYB148, and other MYB transcription factors involved in phenylpropanoid metabolism was constructed using MAFFT^8^ and MEGA5.0 software (**Figure 4**). The results indicated that transcription factors with similar functions formed separate branches. Thus, ZmMYB111 may function similarly to AtMYB85 (i.e., in the regulation of lignin synthesis), since they were on the same branch, and alternatively, ZmMYB148 may function similarly to Zm1 (i.e., in anthocyanin biosynthesis), since they were on the same branch. Conserved motif analysis of ZmMYB111, ZmMYB148, and the other MYB transcription factors using the MEME software^9^ showed that both ZmMYB111 and ZmMYB148 shared three motifs (motif 1, motif 2, and motif 3) in common with all 21 other MYB domain transcription factors analyzed in this paper (**Figure 5**). In addition, ZmMYB111 also contained motif 4, which is found in AtMYB32, Zm42, Zm31, and AtMYB85, all of which are involved in lignin synthesis; motif 5, which is found in ZmMYB-IF35, ZmMYB-IF25, AtMYB/PAP1, and AtMYB/PAP2, all of which are involved in flavonoid synthesis; and motif 7, which is found in ZmC1, PL2, AtMYB/PAP1, and AtMYB/PAP2, all of which are involved in anthocyanin synthesis (Cone et al., 1993; Lesnick and Chandler, 1998). Of the seven conserved motifs found in ZmMYB148, three (motif 7, motif 10, and motif 12) are also found in Zm1, which is involved in flavonoid biosynthesis, and another (motif 11) is also found in AtMYB32, Zm42, Zm31, and AtMYB63, all of which are involved in lignin biosynthesis (**Figure 5**; Franken et al., 1994; Heine et al., 2007).

### Expression Pattern Analyses

**FIGURE 6:**
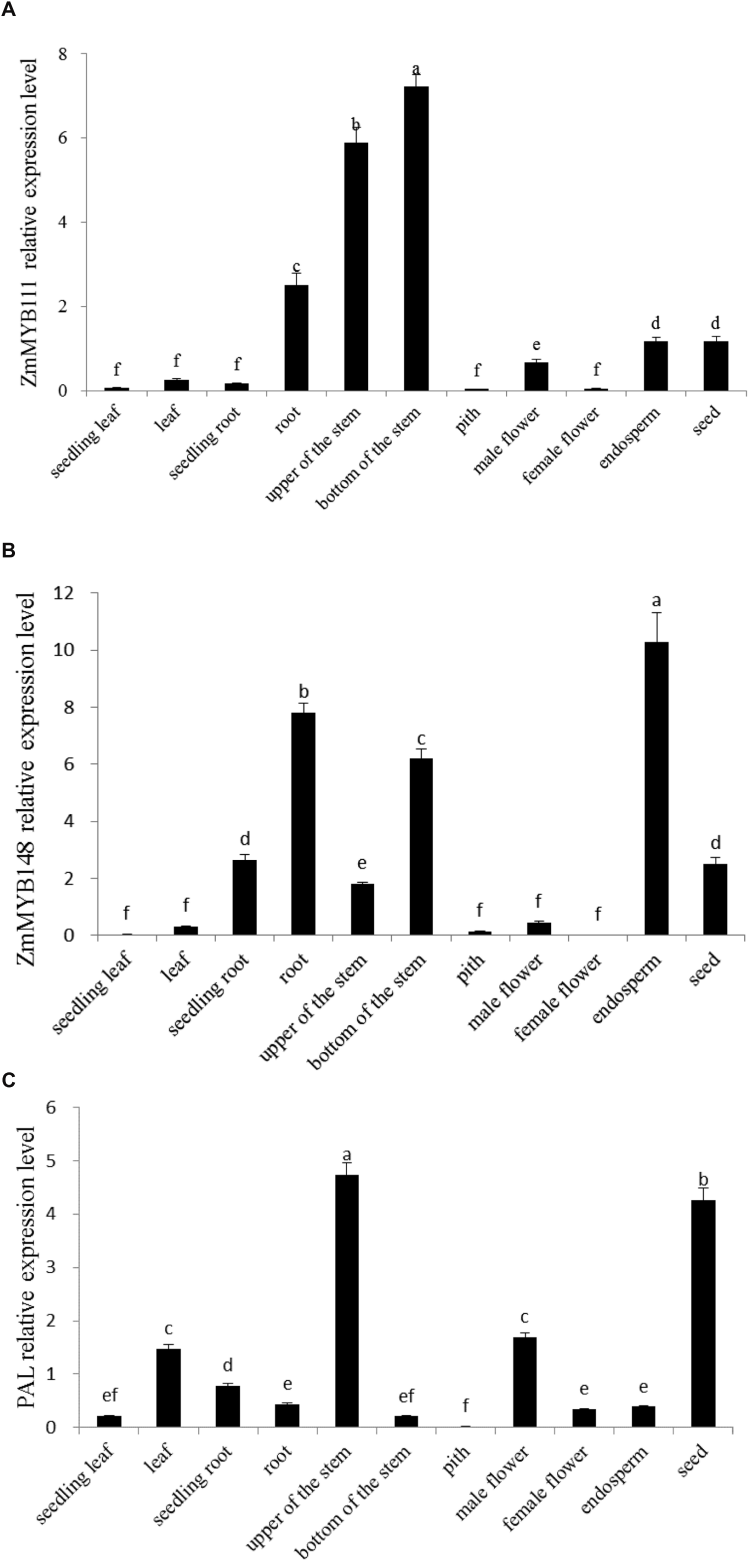
**Expression analysis of ZmMYB111 **(A)**, ZmMYB148 **(B)**, and PAL **(C)** genes in different organs of maize.** Relative expression levels were measured using qRT-PCR. Data represent the mean ± SD of three biological replicate experiments and three technical replicates and were analyzed by Duncan’s test (*n* = 3). Different lower case letter (a–f) indicates the significant difference at *P* ≤ 0.05.

When we examined the expression patterns of ZmMYB111 and ZmMYB148 in different organs of maize plants, we found that ZmMYB111 was expressed in roots, endosperm, seeds, and stems. Furthermore, it was expressed most highly in lower stems and was extremely low in leaves, seedling roots, or male flowers (**Figure 6A**). In contrast, the expression of ZmMYB148 was highest in endosperm, followed by roots and lower stems, and was extremely low in leaves, seedling roots, pith, and female flowers (**Figure 6B**). These results suggest that the ZmMYB111 and ZmMYB148 genes might play an important role in maize endosperm development.

The PAL gene is a key component of phenylpropanoid metabolism, and it may be the target gene of the transcription factor ZmMYB148 (Du et al., 2012); therefore, its expression pattern was also investigated. Our results indicated that the PAL gene was transcribed in maize endosperm (**Figure 6C**). Therefore, we concluded that it could be useful for conducting transient expression assays in maize endosperm in order to measure the regulation functions of ZmMYB111 and ZmMYB148.

### Subcellular Localization

**FIGURE 7:**
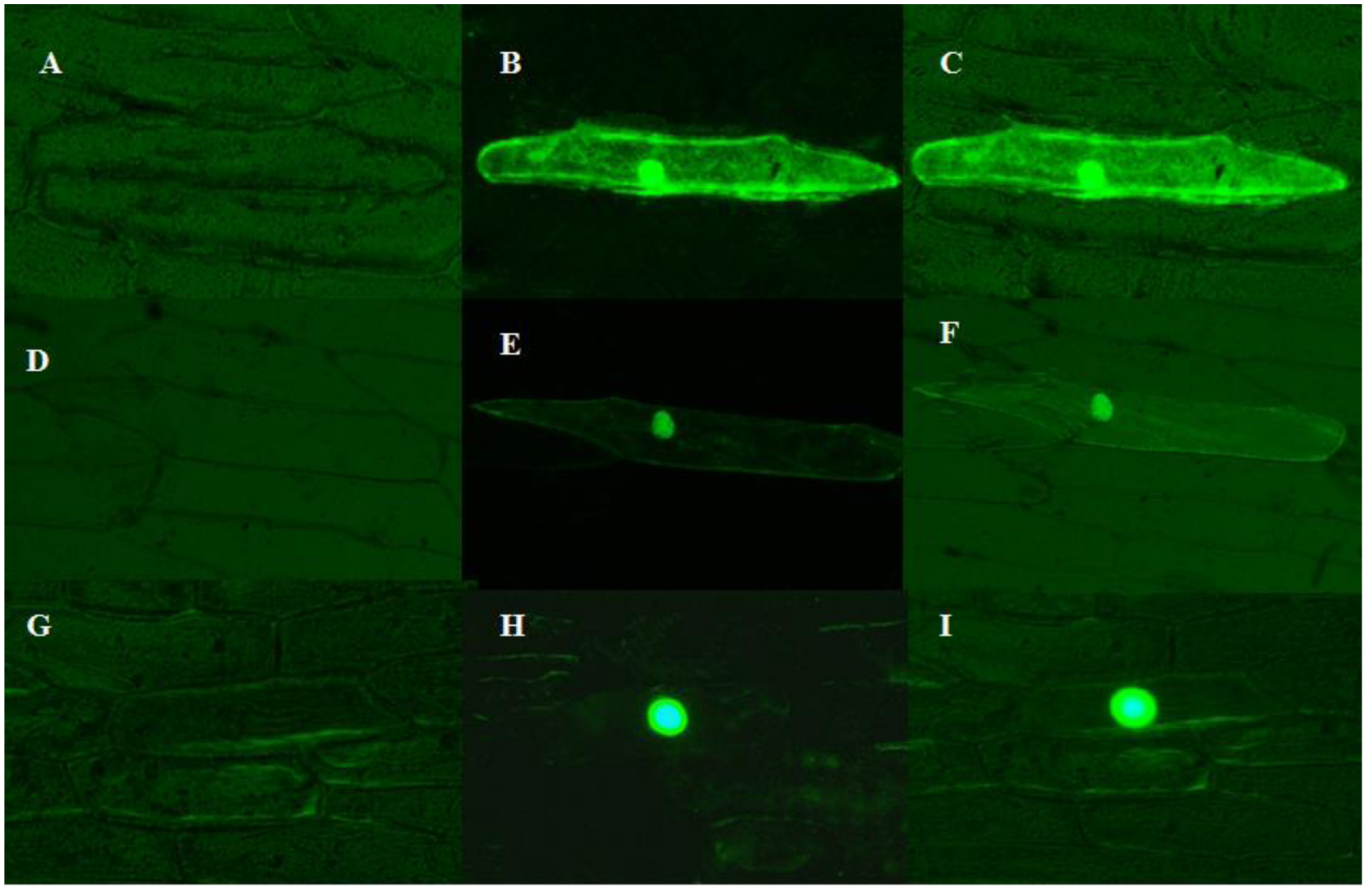
**Subcellular localization of ZmMYB111 and ZmMYB148.** The fusion protein, driven by the 35S promoter, was transiently expressed in onion epidermal cells and analyzed by fluorescent microscopy. GFP alone was used as a control. **(A)** p2300-35S-eGFP image under white light. **(B)** p2300-35S-eGFP image under blue light. **(C)** p2300-35S-eGFP image under blue light and white light. **(D)** eGFP- ZmMYB111 image under white light. **(E)** eGFP- ZmMYB111 image under blue light. **(F)** eGFP- ZmMYB111 image under blue light and white light. **(G)** eGFP- ZmMYB148 image under white light. **(H)** eGFP- ZmMYB148 image under blue light. **(I)** eGFP- ZmMYB148 image under blue light and white light.

Most transcription factors localized to the nucleus. The subcellular localization of ZmMYB111 and ZmMYB148 proteins was determined in onion epidermal cells. As shown in **Figure 7**, both ZmMYB111 and ZmMYB148 were located in the nucleus, whereas the control GFP was localized in both cytoplasm and the nucleus.

### Transcription Activation

**FIGURE 8:**
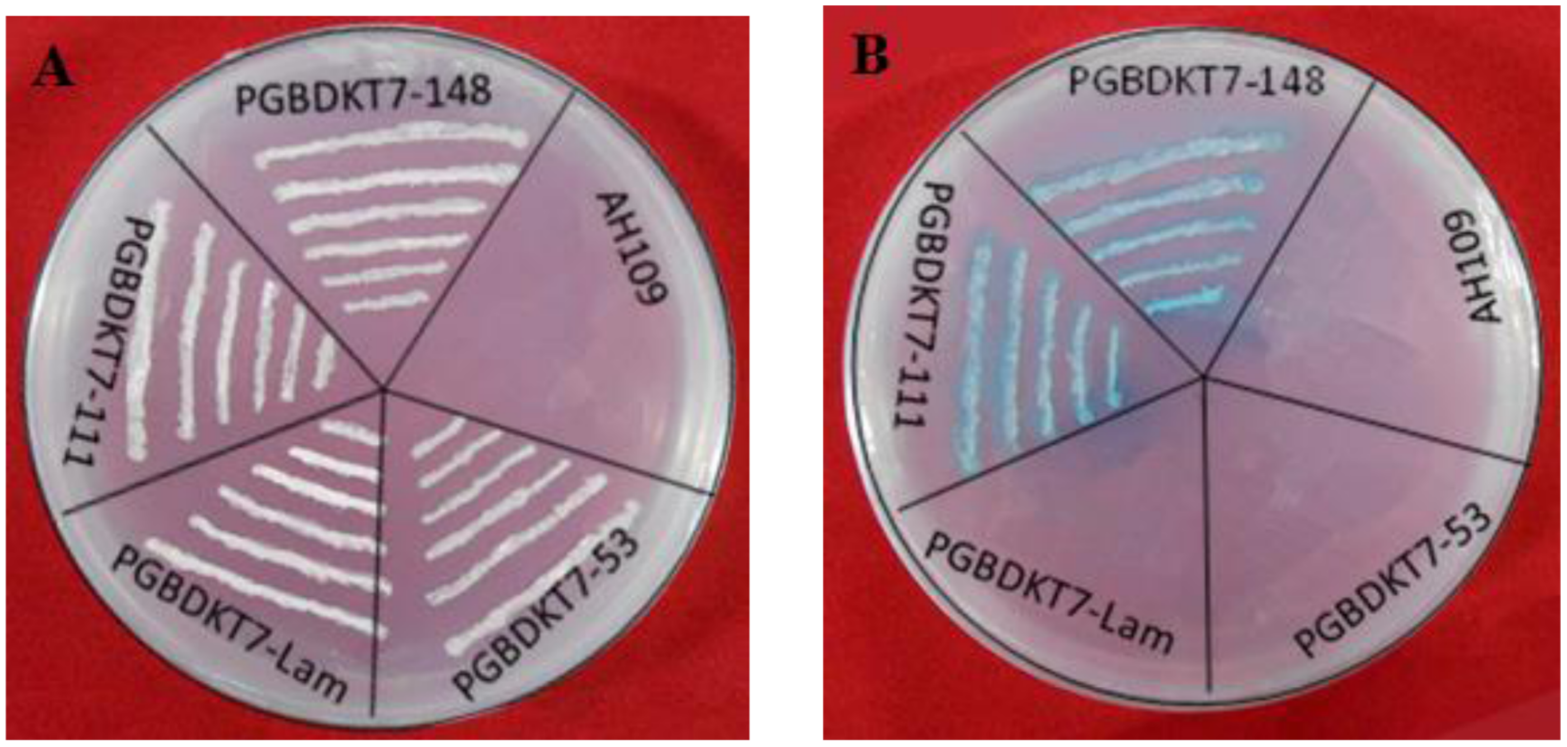
**Transactivation activity assays of ZmMYB148 and ZmMYB111 in yeast.** AH109, yeast strain AH109 without any vector; PGBDKT7-Lam and PGBDKT7-53, negative controls. The transformants were screened on SD/-Trp medium **(A)**. The colonies were screened on SD/-Trp-His-Leu medium with X-α-gal **(B)**.

The constructs of pGBKT7-ZmMYB111 and pGBKT7-ZmMYB148 were separately transformed into yeast AH109 cells and screened on SD/-Trp medium. Positive clones were identified using PCR and were further cultivated on SD/-Trp/-His/10mmol3-AT/X-α-gal mediums. We found that the yeast cells transformed with pGBKT7-ZmMYB111 and pGBKT7-ZmMYB148 were able to grow and change the color of the medium to blue, which suggests that both ZmMYB111 and ZmMYB148 possess transcription activation activities (**Figure 8**).

### Protein–DNA Interaction

Phenylalanine ammonia-lyase and 4CL are the key enzymes in phenylpropanoid metabolism. Promoters of the PAL and 4CL genes were amplified from maize B73 gDNA using PCR and analyzed by agarose gel electrophoresis. Sequencing results revealed that the promoter sequences were consistent with GenBank report, and the lengths of the promoters were 1867 and 1873 bp, respectively. The promoters were separately ligated into the pHis2 plasmid to generate the pHis2-pPAL and pHis2-p4CL constructs that will be used for yeast one-hybrid screening for DNA-protein interactions.

**FIGURE 9:**
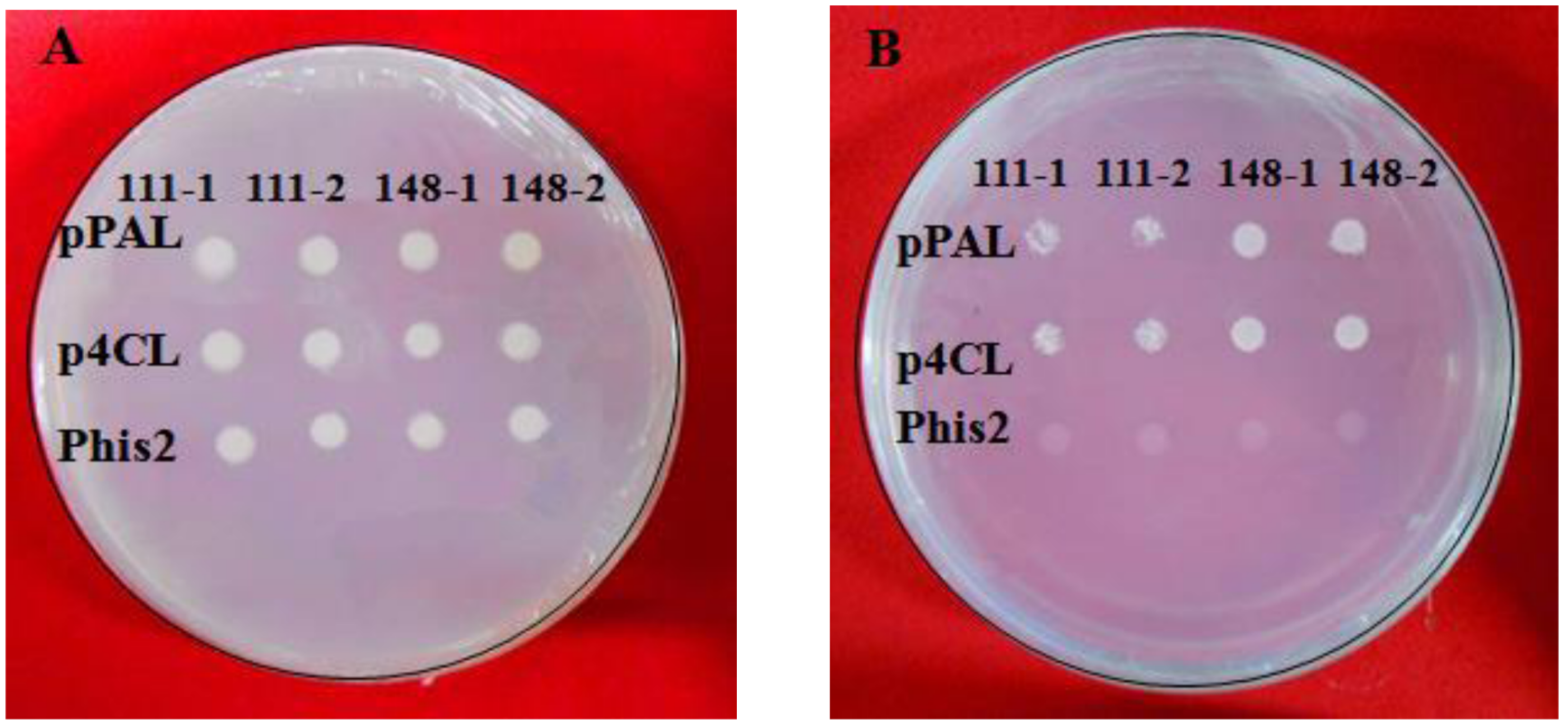
**Binding characteristics of ZmMYB111 and ZmMYB148 transcription factors with the promoter of PAL and 4CL using yeast one-hybrid analysis.** The related recombinant expression vectors were co-transformed into the yeast strain Y187, and empty pHis2 was used as a negative control. **(A)** The growth status of the transformed yeasts on SD/-Trp/-Leu medium. **(B)** The growth status of the transformed yeasts on SD/-Leu-Ura-His+ medium with 10 mM 3-AT.

The constructs were co-transformed into the yeast strain Y187, and the yeast cells were screened using a synthetic dropout nutrient medium. As is shown in **Figure 9**, yeast transformants that contained the plasmid pGADT7-ZmaMYB111/pGADT7-ZmaMYB148 and pHis2-pPAL or pGADT7-ZmaMYB111/pGADT7-ZmaMYB148 and pHis2-p4CL were able to grow on the SD/-Trp/-Leu and SD/-Trp/-Leu/-His/100mmol3-AT mediums. However, the yeast transformants harboring pHis2 could only grow on the SD/-Trp/-Leu medium (**Figure 9**). These results suggest that both ZmaMYB111 and ZmMYB148 are able to bind to the promoters of the PAL and 4CL genes and may regulate their transcription.

### Transient Expression Assays in Maize Endosperm

**FIGURE 10:**
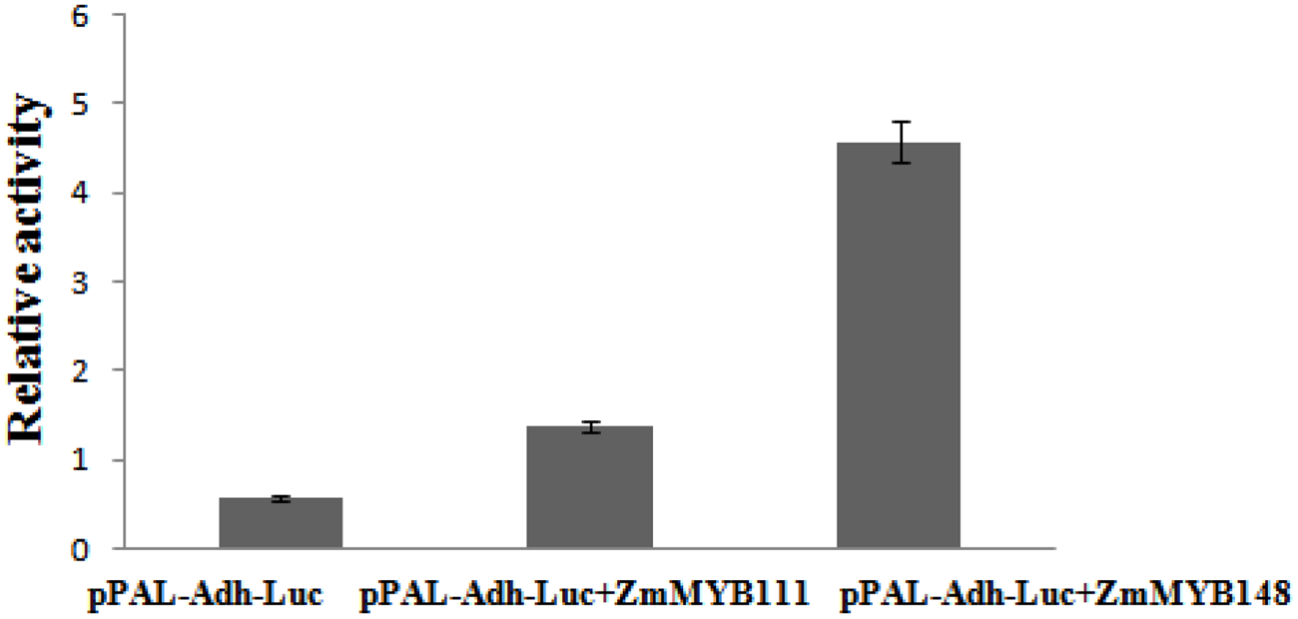
**Phenylalanine ammonia-lyase (PAL) promoter activity, induced by the transcription factors ZmMYB111 (middle) and ZmMYB148 (right) in our transient expression experiment.** The related constructs were co-transformed into 9 DAP endosperm, and empty pBI221 was used as a control (left) in order to correct for transfection efficiency. The activities of GUS and LUC were measured, and the activity ratio of LUC/GUS was calculated to reflect the relative activity of the PAL promoter.

The relative constructs were co-transformed into maize endosperms in order to analyze the binding characteristics of ZmaMYB111 and ZmMYB148 transcription factors to the PAL promoter. As shown in **Figure 10**, the activity ratio of LUC/GUS was only 0.5 in the case of the pBI221-pPAL-ADH. However, in the presence of pBI221-ZmaMYB111 and pBI221-ZmaMYB148, the ratio of LUC/GUS reached 1.6 and 4.8 and increased by 3 and 8 times, respectively (**Figure 10**). The results indicate that the ZmMYB111 and ZmMYB148 transcription factors are able to bind to the PAL promoter and improve the expression of the PAL gene.

## Discussion

Phenylpropanoid metabolism plays an important role in crop resistance to abiotic and biotic stressors, as well as in quality (Richard and Paiva, 1995; Payyavula et al., 2012). The process begins when L-phenylalanine is transformed to *t*-cinnamic acid by PAL, the activity of which is regulated at the transcriptional level, depending on the stage of cell differentiation and exposure to various kinds of stress (Weitzel and Petersen, 2010). In addition, PAL is an important branch point enzyme that links primary and secondary plant metabolism, including the production of anthocyanins, flavonoids, monolignols, coumarins, etc. (Weitzel and Petersen, 2010). In this paper, the ZmMYB111 and ZmMYB148 transcription factors demonstrated clear regulatory influence on the transcription of the PAL gene, so it is possible that they are also involved in regulating the synthesis of phenylpropanoid compounds.

Indeed, a number of MYB transcription factors have been confirmed in the regulation of flavonoid and lignin biosynthesis in *Arabidopsis*. For example, the transcription factors MYB58, MYB46, MYB63, MYB75, and MYB83 have all been shown to be involved in the regulation of lignin synthesis (Zhou et al., 2009; Bhargava et al., 2010; Ko et al., 2014), and MYB12, MYB75, MYB/PAP1, and AtMYB/PAP2 are known to be involved in flavonoid synthesis (Cone et al., 1993; Lesnick and Chandler, 1998; Nakabayashi et al., 2014). Furthermore, in maize, the MYB31 and MYB42 transcription factors were shown to inhibit lignin synthesis (Sonbol et al., 2009; Fornalé et al., 2010), ZmC1 was shown to regulate anthocyanin synthesis (Franken et al., 1994), and MYB-IF35 was shown to be involved in flavonoid synthesis (Heine et al., 2007).

In general, fewer MYB transcription factors have been described as involved in phenylpropanoid metabolism in maize, and even those have mainly been involved in the regulation of downstream reactions. In contrast, our study suggests that the ZmMYB111 and ZmMYB148 transcription factors might regulate the first key enzyme gene (the PAL gene) simultaneously, and ZmMYB111 and ZmMYB148 might be involved in the synthesis of both flavonoids and lignins. Accordingly, we speculated that the ZmMYB111 and ZmMYB148 transcription factors could have a much broader application in maize breeding.

In our study, RT-qPCR analysis indicated that the expressions of ZmMYB111 and ZmMYB148 in leaves, roots, and stems were higher in mature plants than in seedlings. In other words, their expression levels were higher in mature tissues than in young tissues of the same plants (**Figures 6A,B**). Interestingly, it was previously reported that R2R3-MYB transcription factors involved in lignin synthesis were mainly expressed in lignified organization, and higher expression levels have been found in mature tissues than in tender tissues (McCarthy et al., 2011; Handakumbura and Hazen, 2012), which is consistent with our results. Bioinformatics analysis also revealed that ZmMYB111 and ZmMYB148 were associated with lignin synthesis. Thus, we speculate that the ZmMYB111 and ZmMYB148 genes might play a pivotal role in the lignin synthesis of maize.

Expression of the PAL gene has been reported at various levels in all organs of plants (Fock-Bastide et al., 2014; de Jong et al., 2015), and our results also indicated that the PAL gene could be transcribed in all organs of maize, including endosperm, and especially in stems and seeds (**Figure 6C**). Production of stable, fertile transgenic lines is an essential technique in gene function studies; however, transformation in maize is difficult with low transformation rate and long breeding times. Therefore, using transient transformation approaches to study gene function is an alternative method (Reyes et al., 2010; Wu et al., 2014). Because the ZmMYB148 gene was highly expressed in maize endosperm, 9 DAP endosperm was used as the receptor of transient expression, which was conducted in order to determine the regulatory effect of the ZmMYB148 protein on the expression of the PAL gene. Transient expression in maize endosperms is a good method for identifying the roles of target genes expressed in endosperm and has been used successfully in our previous studies (Hu et al., 2012; Zhang et al., 2014). In addition, flavonoids and their derivatives are abundant in mature maize kernels, and they have specified the functions (Koes et al., 2005; Falcone Ferreyra et al., 2012; Wen et al., 2014). In conclusion, we speculate that the ZmMYB148 gene might play a role in the synthesis of flavonoids, based on the results of conserved motifs analysis, the phylogenetic tree, and qPCR. Therefore, the gene may find an application in breeding programs for the development of maize with improved flavonoid content.

## Author Contributions

YH designed and supervised this study. JZ, SZ, Hui Li, Yangping Li, and HH performed the experiments. YH, Hanmei Liu, and GY performed the data analysis. Yinghong Liu performed the fieldwork. JZ designed the experiments and was extensively involved in the writing the manuscript.

## Conflict of Interest Statement

The authors declare that the research was conducted in the absence of any commercial or financial relationships that could be construed as a potential conflict of interest.

The reviewer AM and handling Editor declared their shared affiliation, and the handling Editor states that the process nevertheless met the standards of a fair and objective review.

## Footnotes

1 http://faostat.fao.org/site/339/default.aspx

2 http://www.ncbi.nlm.nih.gov/

3 www.majorbio.com

4 www.majorbio.com

5 http://bioinf.cs.ucl.ac.uk/psipred/

6 http://swissmodel.expasy.org

7 http://prosite.expasy.org/

8 http://mafft.cbrc.jp/alignment/server/index.html

9 http://meme.nbcr.net/meme/
