# Supplementary material for: Identification of Transcription Factors ZmMYB111 and ZmMYB148 Involved in Phenylpropanoid Metabolism
Source: Front Plant Sci. 2016 Feb 15;7:148. doi: 10.3389/fpls.2016.00148 (PMC4753300; doi:10.3389/fpls.2016.00148)
Supplement: Supplementary file 1 [file Data_Sheet_1.DOCX]

**SUPPLEMENT 1** PCR amplification products of *ZmMYB111* (left) *and* *ZmMYB148* (right)*.* 5000M*,* DL5000 DNA Marker.


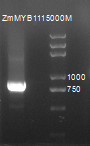

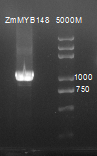


**SUPPLEMENT 2** The cDNA sequence and amino acid sequence of ZmMYB111

ZmMYB111Zmays|GRMZM2G104551|GRMZM2G104551_T01(21,0;314,2)

MGRQPCCDKLGVKRGPWTAEEDRKLINFILTNGHCCWRAVPKLAGLLRCGKSCRLRWTNYLRPDLKRGLLTDAEEQVVIDLHAKLGNRWSKIAAKLPGRTDNEIKNHWNTHIKKKLIKMGIDPVTHEPLDRKTTSSGPATTSQSTKSDEATKEQSPQNDDAVIRDVPADGCSPTESSTNTVSTGGSSSSGGGGHDQDPLVKWLLEEEPATGDEAWLNFTGSVDVDEFSSIAAGPELLPWDGATDWLLDYQDFGLGDSSLVDGYMVNNNSSNGAKF*


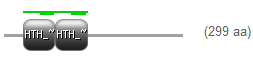

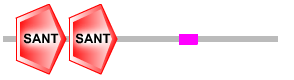


MGRQPCCDKLGVKRGPWTAEEDRKLINFILTNGHCCWRAVPKLAGLLRCGKSCRLRWTNYLRPDLKRGLLTDAEEQVVIDLHAKLGNRWSKIAAKLPGRTDNEIKNHWNTHIKKKLIKMGIDPVTHEPLDRKTTSSGPATTSQSTKSDEATKEQSPQNDDAVIRDVPADGCSPTESSTNTVSTGGSSSSGGGGHDQDPLVKWLLEEEPATGDEAWLNFTGSVDVDEFSSIAAGPELLPWDGATDWLLDYQDFGLGDSSLVDGYMVNNNSSNGAKF*

>GRMZM2G104551_T01CDS

ATGGGTCGGCAGCCGTGCTGCGACAAGCTGGGGGTGAAGCGGGGGCCGTGGACGGCGGAGGAGGACCGCAAGCTCATCAACTTCATCCTCACCAACGGCCATTGCTGCTGGCGCGCGGTGCCCAAGCTCGCCGGCCTGCTGCGCTGCGGCAAGAGCTGCCGCCTGCGCTGGACCAACTACCTCCGCCCGGACCTCAAGCGCGGGCTCCTCACGGACGCCGAGGAGCAGGTCGTCATCGACCTCCACGCCAAGCTCGGCAACAGATGGTCGAAGATTGCTGCCAAGCTACCGGGCAGGACTGACAACGAGATCAAGAACCACTGGAACACGCACATTAAGAAGAAGCTGATCAAGATGGGCATCGATCCAGTCACGCACGAACCCCTCGACCGGAAGACAACCAGCAGCGGCCCGGCTACAACCTCTCAGTCAACCAAGTCTGACGAGGCGACCAAGGAGCAGAGCCCGCAGAACGACGACGCCGTGATAAGGGACGTGCCGGCCGATGGTTGCAGCCCGACGGAATCGAGCACGAACACCGTGAGCACCGGCGGAAGCAGCAGCAGTGGTGGCGGCGGCCATGACCAAGACCCGCTGGTGAAGTGGCTTTTGGAAGAGGAGCCTGCCACCGGCGACGAGGCGTGGCTGAACTTCACTGGCAGTGTCGACGTGGACGAGTTCAGCAGCATTGCCGCCGGTCCGGAGTTGTTGCCGTGGGATGGCGCGACCGACTGGCTGCTCGACTACCAAGATTTTGGATTGGGGGACTCGAGCTTGGTCGATGGCTACATGGTCAACAACAACAGCTCAAACGGAGCAAAGTTCTAG

**SUPPLEMENT 3 The cDNA sequence and amino acid sequence of ZmMYB148**

MGKGRAPCCAKVGLNKGSWTPEEDMRLIAYIQKYGHANWRALPKQAGLLRCGKSCRLRWINYLRPDLKRGNFTAEEEEAIIKLHGLLGNKWSKIASCLPGRTDNEIKNVWNTHLKKRVSPAGEERGAAGSKKKKKKKTTKAAAGGGAEAPLPLPSPSPSSSTTTTNFSSGDSGEQQSNNMSKEADDELDLENFEMMPMLDVDDPSFGFGTLVDTAPAPYGSAVSVSASAATSPCASSTSPPPASAPPGVDDLLVLPEIDMGHELWSIIDGDAAEAPAPRCQRNPAEPTNGADAGSHGAEGKEWWLEDLERELGLWGTVEDYQYPMGPQGLLVADHPDPLPAMVDDPVSCYFQAGPASAVLQELPGYPVPATAVTGSINQMGL*

MGKGRAPCCAKVGLNKGSWTPEEDMRLIAYIQKYGHANWRALPKQAGLLRCGKSCRLRWINYLRPDLKRGNFTAEEEEAIIKLHGLLGNKWSKIASCLPGRTDNEIKNVWNTHLKKRVSPAGEERGAAGSKKKKKKKTTKAAAGGGAEAPLPLPSPSPSSSTTTTNFSSGDSGEQQSNNMSKEADDELDLENFEMMPMLDVDDPSFGFGTLVDTAPAPYGSAVSVSASAATSPCASSTSPPPASAPPGVDDLLVLPEIDMGHELWSIIDGDAAEAPAPRCQRNPAEPTNGADAGSHGAEGKEWWLEDLERELGLWGTVEDYQYPMGPQGLLVADHPDPLPAMVDDPVSCYFQAGPASAVLQELPGYPVPATAVTGSINQMGL*


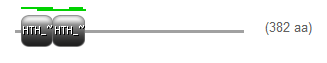

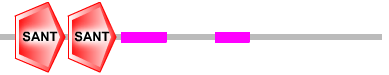


>GRMZM2G097636_T01 CDS
ATGGGGAAGGGCCGGGCACCGTGCTGCGCCAAGGTGGGGCTCAACAAAGGCTCCTGGACGCCGGAAGAGGACATGCGCCTCATCGCCTACATTCAGAAGT
ACGGCCACGCCAACTGGCGCGCCCTGCCCAAGCAAGCAGGTCTGCTGCGGTGCGGGAAGAGCTGCCGGCTGCGGTGGATCAACTACCTGCGCCCGGACCT
CAAGCGCGGCAACTTCACCGCCGAGGAGGAGGAGGCCATCATCAAGCTCCACGGCTTGCTCGGGAACAAGTGGTCCAAGATCGCGTCGTGCCTGCCGGGC
CGGACGGACAACGAGATCAAGAACGTCTGGAACACGCACTTGAAGAAGCGGGTGTCGCCGGCCGGGGAGGAGCGCGGCGCCGCCGGGTCCAAGAAGAAGA
AGAAAAAGAAGACGACGAAGGCAGCCGCTGGCGGTGGCGCGGAGGCGCCGCTGCCGCTCCCGTCGCCGTCGCCGTCCTCGTCCACGACGACGACCAACTT
CTCCAGCGGCGACTCCGGCGAGCAGCAGAGCAACAACATGAGCAAGGAAGCGGACGACGAGCTGGACCTGGAGAACTTCGAGATGATGCCGATGCTCGAC
GTCGACGACCCCAGCTTCGGCTTCGGCACGCTGGTGGACACCGCGCCAGCGCCGTACGGCTCGGCCGTGTCCGTGTCCGCGTCCGCGGCAACGTCGCCGT
GCGCCTCGTCCACGTCCCCGCCGCCGGCGAGCGCCCCGCCCGGCGTGGACGACCTGCTCGTGCTGCCCGAGATCGACATGGGCCATGAGCTGTGGAGCAT
CATCGACGGCGACGCCGCCGAGGCACCGGCGCCGCGCTGCCAGAGGAATCCGGCAGAGCCGACGAACGGCGCCGACGCGGGCAGCCACGGAGCGGAGGGA
AAGGAGTGGTGGTTGGAGGACCTGGAGAGGGAGTTGGGCCTGTGGGGCACCGTGGAGGACTACCAGTACCCAATGGGCCCCCAGGGTCTGCTGGTCGCTG
ACCACCCGGACCCGCTTCCTGCCATGGTGGACGACCCAGTGTCCTGCTACTTCCAGGCGGGCCCCGCCTCGGCCGTGCTCCAGGAACTACCCGGATACCC
TGTCCCTGCTACTGCAGTCACAGGCAGTATTAATCAGATGGGGTTGTGA

**SUPPLEMENT4** The amino acid sequences of the related transcription factors for building phylogenetic tree

>ZmMYB-IF35

MGRAPCCEKVGLKKGRWTKEEDEVLARYIKEHGEGSWRSLPKNAGLLRCGKSCRLRWINYLRAGLKRGNISEEEEDMIIKLHATLGNRWSLIAGHLPGRTDNEIKNYWNSHLSRRAADFRDGVVVDIDLSKLPGGGKRRGGRASRGAVVAAAKEKKAKEKDDRGNSKVAEAEQQLRDTEDDDGGSVSTPRPQSDDCGTAQSEEEQAQASASGLTSDGHGPEEEEEEDPLALSEEMVSALLAPESPKLEVGPDGSCMDSYSGPPSGESGCGSSGPSGDVAQDLDLDDDKAIMDWDLMGLDISTAGDMWDQLVWDYDETLVTEPEGGEEGHQQQDDVMSDLFFLDNL

>ZmMYB-IF25

MGRAPCCGKVGLKKGRWTREEDEILARYIEEHGEGSWRSLPKNAGLLRCGKSCRLRWINYLRAGLKRGNITEEEEDVIVKLHATLGNRWSLIAGHLPGRTDNEIKNHWNSHLRRRGRAGDSRDDGVVVNIDLVKLPGGRRASSRGIVAAAKPAGKNKEKRKNGKGRNNVAEAEQQLEEEDANVSTTTPRTQSHCASAAQSEEQAQASASGLTSEEDLLALSEEMVVSALLAPGSPKLLEVGPDGSCVDGDGGLSGDSGRGSGGPSGDVAQELHLDDDAIMDWDSMGLDIPTADDTWNPLVWDYDQTSLVPEPEGEGRRQRDEMMSDLFFLDNL

>ZmC1

MGRRACCAKEGVKRGAWTSKEDDALAAYVKAHGEGKWREVPQKAGLRRCGKSCRLRWLNYLRPNIRRGNISYDEEDLMIIRLHRLLGNRWSLIAGRLPGRTDNEIKNYWNSTLGRRAGAGAGAGGSRVVIAPDTGSHATPAATSGSGETGQKGAAPRADPDSAGTTTTSAAAVWAPKAVRCTGGLFFFHRDTTPAHAGETATPMAGGGLGGEAGSSEDCSSAASVSPLVG

SQDEPCFSGDGDCDWMDDVRALASFLESDEDWLRCQTAGQLA

>PL

MGRRACCAKEGVKRGAWTAKEDDTLAAYVKAHGEGKWREVPQKAGLRRCGKSCRLRWLNYLRPNIKRGNISYDEEDLIVRLHKLLGNRWSLIAGRLPGRTDNEIKNYWNSTLGRRAGAGAGAGGSRVVFAPDTGSHATPAAAGSREMTGGQKGAAPRADLGSPGSAAVVWAPKAARCTGGLFFHRRDTHTPHAGGTETPTPMMAGGAGGEARSSDDCSSAASVSVSPLVGSSQHDPCFSGDGNGDWMDDVRALASFLESDEEWLRCHTAEQLV

>P1

MGRAPCCEKVGLKRGRWTAEEDQLLANYIAEHGEGSWRSLPKNAGLLRCGKSCRLRWINYLRADVKRGNISKEEEDIIIKLHATLGNRWSLIASHLPGRTDNEIKNYWNSHLSRQIHTYRRKYTAGPDDTAIAIDMSKLQSADRRRGGRTPGRPPKASASRTKQADADQPGGEAKGPAAAASSPRHSDVVNPGPNQPNSSSGSTGTAEEEGPSSEDASGPWVLEPIELGDLVWGEADSEMDALMPIGPGGHDSAALEGLGAVGCEAQVDDLFDMDWDGFAAHLWGGPEQDEHSAQLRQAAEPLEVAAAAAAATAARTPDDRELEAFETWLLSDSF

>P2

MGRAPCCEKVGLKRGRWTAEEDQLLANYIAEHGEGSWRSLPKNAGLLRCGKSCRLRWINYLRADVKRGNISKEEEDIIIKLHATLGNRWSLIASHLPGRTDNEIKNYWNSHLSRQIHTYRRKYTAGPDDTAIAIDMSKLQSADRRRGGRTPGRPPKTSASRTKHSDADQPGGEAKGAAASSPRHSDAVNPGPNQPNSSSGSTGTAEEEGPSSEDASGPWVLEPIELGDLWGEADSEMDALMPIGPGGHDSAALQGLGAVGGEAQVDDLFDMDWDGFAAHLWGGPEQDDHSAQLRQAAEPMEAAAVAAAAAAATAACTPDDRELEAFETWLLSDSF

>Zm42

MGRSPCCEKAHTNRGAWTKEEDERLVAYVRAHGEGCWRSLPRAAGLLRCGKSCRLRWINYLRPDLKRGNFTADEDDLIVKLHSLLGNKWSLIAARLPGRTDNEIKNYWNTHIRRKLLGSGIDPVTHRRVAGGAATTISFQPSPNTAVAAAAETAAQAPIKAEETAAVKAPRCPDLNLDLCISPPCQHEDDGEEEEEELDLIKPAVVKREALQAGHGHGHGLCLGCGLGGQKGAAGCSCSNGHHFLGLRTSVLDFRGLEMK

>Zm31

MGRSPCCEKAHTNKGAWTKEEDERLVAHIRAHGEGCWRSLPKAAGLLRCGKSCRLRWINYLRPDLKRGNFTEEEDELIVKLHSVLGNKWSLIAGRLPGRTDNEIKNYWNTHIRRKLLSRGIDPVTHRPVTEHHASNITISFETEVAAAARDDKKGAVFRLEEEEERNKATMVVGRDRQSQSQSHSHPAGEWGQGKRPLKCPDLNLDLCISPPCQEEEEMEEAAMRVRPAVKREAGLCFGCSLGLPRTADCKCSSSSFLGLRTAMLDFRSLEMK

>Zm148

MGKGRAPCCAKVGLNKGSWTPEEDMRLIAYIQKYGHANWRALPKQAGLLRCGKSCRLRWINYLRPDLKRGNFTAEEEEAIIKLHGLLGNKWSKIASCLPGRTDNEIKNVWNTHLKKRVSPAGEERGAAGSKKKKKKKTTKAAAGGGAEAPLPLPSPSPSSSTTTTNFSSGDSGEQQSNNMSKEADDELDLENFEMMPMLDVDDPSFGFGTLVDTAPAPYGSAVSVSASAATSPCASSTSPPPASAPPGVDDLLVLPEIDMGHELWSIIDGDAAEAPAPRCQRNPAEPTNGADAGSHGAEGKEWWLEDLERELGLWGTVEDYQYPMGPQGLLVADHPDPLPAMVDDPVSCYFQAGPASAVLQELPGYPVPATAVTGSINQMGL

>Zm111

MGRQPCCDKLGVKRGPWTAEEDRKLINFILTNGHCCWRAVPKLAGLLRCGKSCRLRWTNYLRPDLKRGLLTDAEEQVVIDLHAKLGNRWSKIAAKLPGRTDNEIKNHWNTHIKKKLIKMGIDPVTHEPLDRKTTSSGPATTSQSTKSDEATKEQSPQNDDAVIRDVPADGCSPTESSTNTVSTGGSSSSGGGGHDQDPLVKWLLEEEPATGDEAWLNFTGSVDVDEFSSIAAGPELLPWDGATDWLLDYQDFGLGDSSLVDGYMVNNNSSNGAKF

>AtMYB12

MGRAPCCEKVGIKRGRWTAEEDQILSNYIQSNGEGSWRSLPKNAGLKRCGKSCRLRWINYLRSDLKRGNITPEEEELVVKLHSTLGNRWSLIAGHLPGRTDNEIKNYWNSHLSRKLHNFIRKPSISQDVSAVIMTNASSAPPPPQAKRRLGRTSRSAMKPKIHRTKTRKTKKTSAPPEPNADVAGADKEALMVESSGAEAELGRPCDYYGDDCNKNLMSINGDNGVLTFDDDIIDLLLDESDPGHLYTNTTCGGDGELHNIRDSEGARGFSDTWNQGNLDCLLQSCPSVESFLNYDHQVNDASTDEFIDWDCVWQEGSDNNLWHEKENPDSMVSWLLDGDDEATIGNSNCENFGEPLDHDDESALVAWLLS

>AtMYB11

MGRAPCCEKVGIKKGRWTAEEDRTLSDYIQSNGEGSWRSLPKNAGLKRCGKSCRLRWINYLRSDIKRGNITPEEEDVIVKLHSTLGTRWSTIASNLPGRTDNEIKNYWNSHLSRKLHGYFRKPTVANTVENAPPPPKRRPGRTSRSAMKPKFILNPKNHKTPNSFKANKSDIVLPTTTIENGEGDKEDALMVLSSSSLSGAEEPGLGPCGYGDDGDCNPSINGDDGALCLNDDIFDSCFLLDDSHAVHVSSCESNNVKNSEPYGGMSVGHKNIETMADDFVDWDFVWREGQTLWDEKEDLDSVLSRLLDGEEMESEIRQRDSNDFGEPLDIDEENKMAAWLLS

>AtMYB111

MGRAPCCEKIGLKRGRWTAEEDEILTKYIQTNGEGSWRSLPKKAGLLRCGKSCRLRWINYLRRDLKRGNITSDEEEIIVKLHSLLGNRWSLIATHLPGRTDNEIKNYWNSHLSRKIYAFTAVSGDGHNLLVNDVVLKKSCSSSSGAKNNNKTKKKKKGRTSRSSMKKHKQMVTASQCFSQPKELESDFSEGGQNGNFEGESLGPYEWLDGELERLLSSCVWECTSEEAVIGVNDEKVCESGDNSSCCVNLFEEEQGSETKIGHVGITEVDHDMTVEREREGSFLSSNSNENNDKDWWVGLCNSSEVGFGVDEELLDWEFQGNVTCQSDDLWDLSDIGEITLE

>OsC1

MGRRACCAKEGMKRGAWTSNEDDVLASYIKSHGEGKWREVPQRAGLRRCGKSCRLRWLNYLRPNIKRGNIDDDEEELIVRLHTLLGNRWSLIAGRLPGRTDNEIKNSRSAARSAPPPPPPPAAAVAARRTPPERRTRRRPAPSCRRASSSSQPPAPTPTQQRQRRRRRRRRPPCGRPRPCGARAGSSSTTVKRRRSPRRRRRRQGN

>AtMYB85

MGRQPCCDKLGVKKGPWTVEEDKKLINFILTNGHCCWRALPKLAGLRRCGKSCRLRWTNYLRPDLKRGLLSHDEEQLVIDLHANLGNKWSKIASRLPGRTDNEIKNHWNTHIKKKLLKMGIDPMTHQPLNQEPSNIDNSKTIPSNPDDVSVEPKTTNTKYVEISVTTTEEESSSTVTDQNSSMDNENHLIDNIYDDDELFSYLWSDETTKDEASWSDSNFGVGGTLYDHNISGADADFPIWSPERINDEKMFLDYCQDFGVHDFGF

>AtMYB75/PAP1

MEGSSKGLRKGAWTTEEDSLLRQCINKYGEGKWHQVPVRAGLNRCRKSCRLRWLNYLKPSIKRGKLSSDEVDLLLRLHRLLGNRWSLIAGRLPGRTANDVKNYWNTHLSKKHEPCCKIKMKKRDITPIPTTPALKNNVYKPRPRSFTVNNDCNHLNAPPKVDVNPPCLGLNINNVCDNSIIYNKDKKKDQLVNNLIDGDNMWLEKFLEESQEVDILVPEATTTEKGDTLAFDVDQLWSLFDGETVKFD

>AtMYB90/PAP2

MEGSSKGLRKGAWTAEEDSLLRLCIDKYGEGKWHQVPLRAGLNRCRKSCRLRWLNYLKPSIKRGRLSNDEVDLLLRLHKLLGNRWSLIAGRLPGRTANDVKNYWNTHLSKKHESSCCKSKMKKKNIISPPTTPVQKIGVFKPRPRSFSVNNGCSHLNGLPEVDLIPSCLGLKKNNVCENSITCNKDDEKDDFVNNLMNGDNMWLENLLGENQEADAIVPEATTAEHGATLAFDVEQLWSLFDGETVELDQSL

>AtMYB4

MGRSPCCEKAHTNKGAWTKEEDERLVAYIKAHGEGCWRSLPKAAGLLRCGKSCRLRWINYLRPDLKRGNFTEEEDELIIKLHSLLGNKWSLIAGRLPGRTDNEIKNYWNTHIRRKLINRGIDPTSHRPIQESSASQDSKPTQLEPVTSNTINISFTSAPKVETFHESISFPGKSEKISMLTFKEEKDECPVQEKFPDLNLELRISLPDDVDRLQGHGKSTTPRCFKCSLGMINGMECRCGRMRCDVVGGSSKGSDMSNGFDFLGLAKKETTSLLGFRSLEMK

>AtMYB32

MGRSPCCEKDHTNKGAWTKEEDDKLISYIKSHGEGCWRSLPRSAGLQRCGKSCRLRWINYLRPDLKRGNFTLEEDDLIIKLHSLLGNKWSLIATRLPGRTDNEIKNYWNTHVKRKLLRRGIDPATHRPINKTPQDSSDSSKTEDSLVKILSFGPQLEKIANFGDERNEKEVMCQKERVEYSVVEERCLDLNLELRISPPWQDQLHDEKNLRFGRVKRMCTACRFGFGNGKECSCDNTKSQTEDSSSSSYSSTDFSSSIGYDFLGLNNRVLDFSTLEMK

>AtMYB58

MGKGRAPCCDKSKVKRGPWSQDEDFKLISFIHKYGHENWRSLPKQAGLLRCGKSCRLRWINYLRPDVKRGNFTAEEEETIIKLHQSFGNKWSKIASKLPGRTDNEIKNVWHTHLKKRLSSKTNLNVDEAASKGSLNEEENSQESSPNASMSFAGSNISSKDDDAQISQKTFEHILTFSDFTEMLQEVDKPELLEMTFDIDPDIWSFIDGSDSSFQQPENRALQESEEDEVDKWFKHLECELGLEENDNQQQQQQHKQGTEDENSSSLLESYELLIH

>AtMYB63

MGKGRAPCCDKTKVKRGPWSPEEDIKLISFIQKFGHENWRSLPKQSGLLRCGKSCRLRWINYLRPDLKRGNFTSEEEETIIKLHHNYGNKWSKIASQLPGRTDNEIKNVWHTHLKKRLAQSSGTADEPASPCSSDSVSRGKDDKSSHVEDSLNRETNHRNELSTSMSSGGSNQQDDPKIDELRFEYIEEAYSEFNDIIIQEVDKPDLLEIPFDSDPDIWSFLDTSNSFQQSTANENSSGSRATTEEESDEDEVKKWFKHLESELGLEEDDNQQQYKEEESSSSSLLKNYELMIH

>SlMYB12

MGRTPCCEKVGIKRGRWTAEEDQILTNYIISNGEGSWRSLPKNAGLLRCGKSCRLRWINYLRSDLKRGNITSQEEDIIIKLHATLGNRWSLIAEHLSGRTDNEIKNYWNSHLSRKVDSLRIPSDEKLPKAVVDLAKKGIPKPIKKSSISRPKNKKSNLLEKEALCCTNMPACDSAMELMQEDLAKIEVPNSWAGPIEAKGSLSSDSDIEWPRLEEIMPDVVIDDEDKNTNFILNCFREEVTSNNVGNSYSCIEEGNKKISSDDEKIKLLMDWQDNDELVWPTLPWELETDIVPSWPQWDDTDTNLLQNCTNDNNNYEEATTMEINNQNHSTIVSWLLS

>AtMYB123/TT2

MGKRATTSVRREELNRGAWTDHEDKILRDYITTHGEGKWSTLPNQAGLKRCGKSCRLRWKNYLRPGIKRGNISSDEEELIIRLHNLLGNRWSLIAGRLPGRTDNEIKNHWNSNLRKRLPKTQTKQPKRIKHSTNNENNVCVIRTKAIRCSKTLLFSDLSLQKKSSTSPLPLKEQEMDQGGSSLMGDLEFDFDRIHSEFHFPDLMDFDGLDCGNVTSLVSSNEILGELVPAQGNLDLNRPFTSCHHRGDDEDWLRDFTC

>PtMYB8

MGRHSCCYKQKLRKGLWSPEEDEKLVRHITKYGHGCWSAVPKQAGLQRCGKSCRLRWINYLRPDLKRGTFSPQEENLIVELHSVLGNRWSQIATHLPGRTDNEIKNLWNSCIKKKLRQRGIDPNTHRPLSEVNAEAGDSKNDNSNKKVETQAAMDESHVSAGNEFKHLNAIPRADTANPKFFHVPVEDNTLIASDSQAMLQNGFINSNSTTTTTTATSTASAANFSLPKEFFLERFNSVNATPTSVEAGFNFINQTTSTQGFTGERDQKLIDNPVLWVLQAPNRSVGFPTENLMPWPGQGLAKAVSDAFSDFSSDVCDYNSIMANPSMYRPGPCLSSLLYSERSLDQDLLDNAGTNCMNGSGAAGSAQYWDNTDNNNNNNVRSSSRSSSCNSSTANLEVNNGAAFGHFWGFGERLDATDITSENERKAPLFLERADQSEYAVKWSEMLPPFSSHTQEETLPIYITTDSKSQDLVSSENINNPNHAALNSAIFPISWQQLQNTEYPVLGDRTATMLSTPLSDPDFHRIAAVLDQI

>Zm1

MGRGRAPCCAKVGLNRGSWTPQEDMRLIAYIQKHGHTNWRALPKQAGLLRCGKSCRLRWINYLRPDLKRGNFTDEEEEAIIRLHGLLGNKWSKIAACLPGRTDNEIKNVWNTHLKKKVAQREKKKAGAGSGDAGTPATAPLSSATSSTTTHNSSGGSDSGDQCGTSREPDATDVCPLQLEDMDVSDMLVDGAPPAAQPMPSPSSSSSLTTCVGGVEELIELPVIDIEPEIWSIIDGESAVARHGGDAAAPCTGTGTAVSTSEAEEAAANDWWLENLEKELGLWGYAEEDTQAHPDLLDHYTGLSPLCALEGDPVSTYFQTGPAAAEPELLVVVEPSAVLL

**SUPPLEMENT 5** Schematic diagram representing the reporter and internal control constructs used in the experiment.


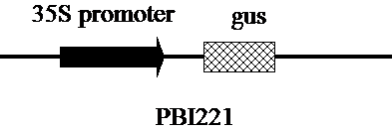


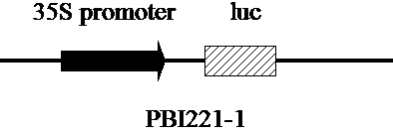


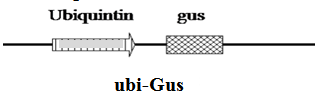


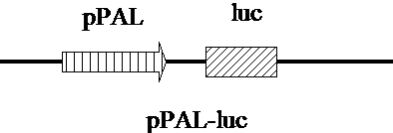


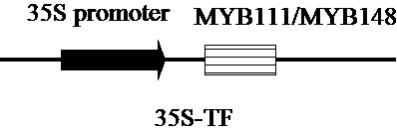


**SUPPLEMENT 6** Total RNA of different organs from maize inbred line B73


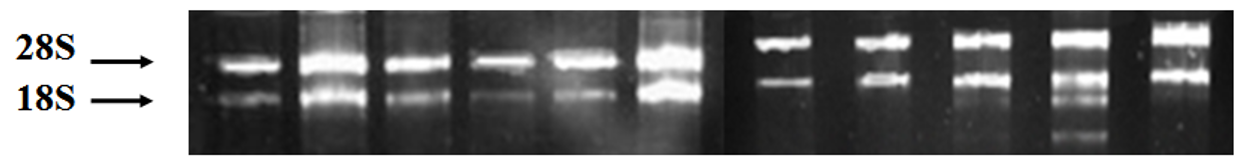


**SUPPLEMENT 7** PCR amplication products of promoters of PAL and 4CL genes. P4CL, the promoter of 4CL gene; pPAL, the promoter of PAL gene; 5000M*，*DL5000 DNA Marker.


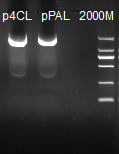


**SUPPLEMENT 8** Standard curve and Melt curve peak chart of ZmMYB111


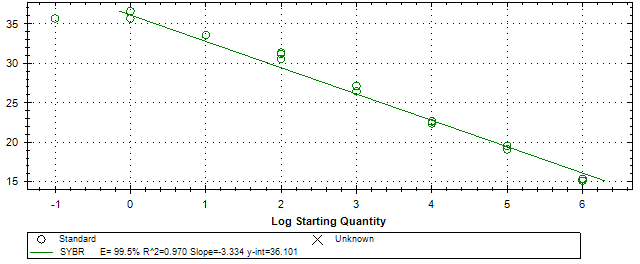


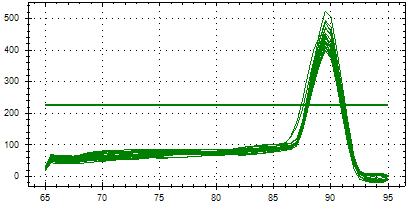


**SUPPLEMENT 9** Standard curve and Melt curve peak chart of ZmMYB148


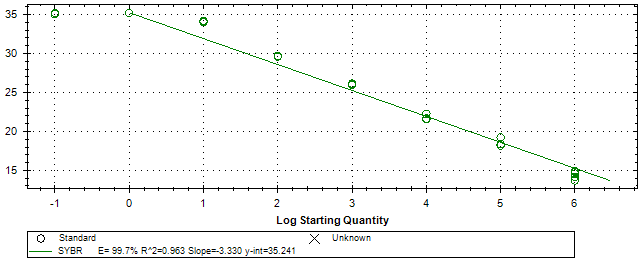


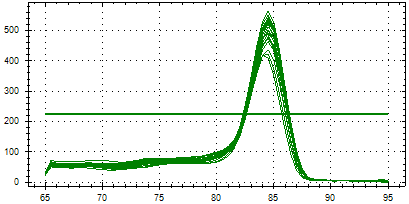


**SUPPLEMENT 10** Standard curve and Melt curve peak chart of PAL gene


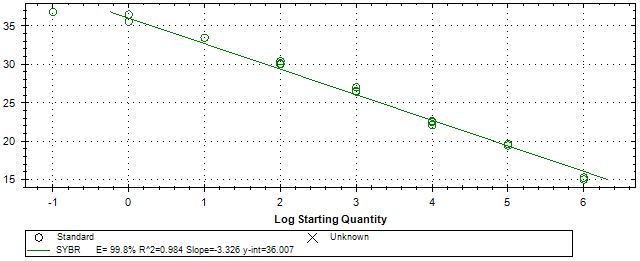

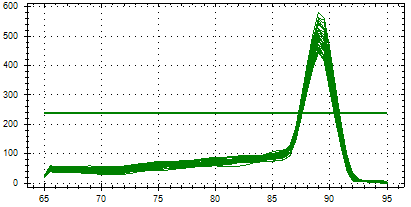


**SUPPLEMENT 11** The diagrams of vectors used in this experiment


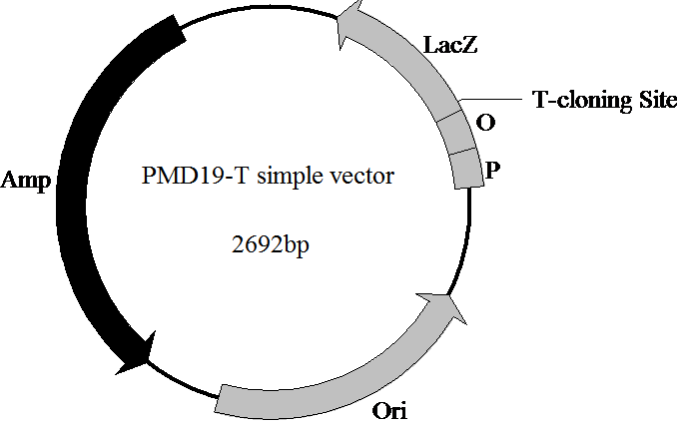

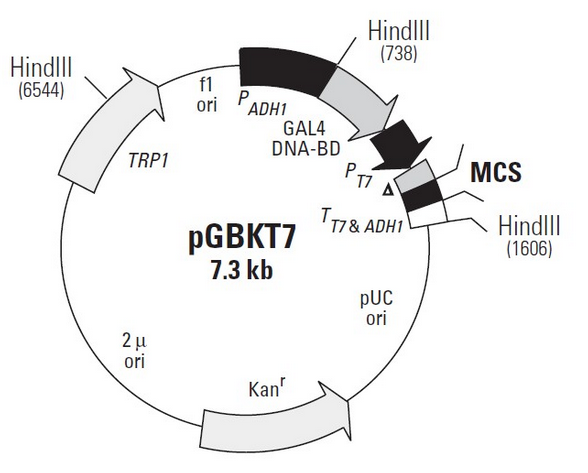

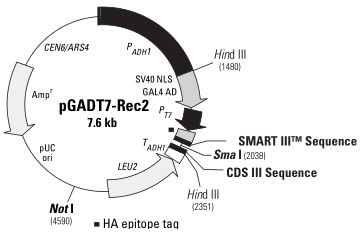

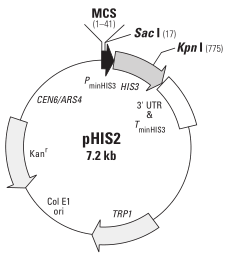


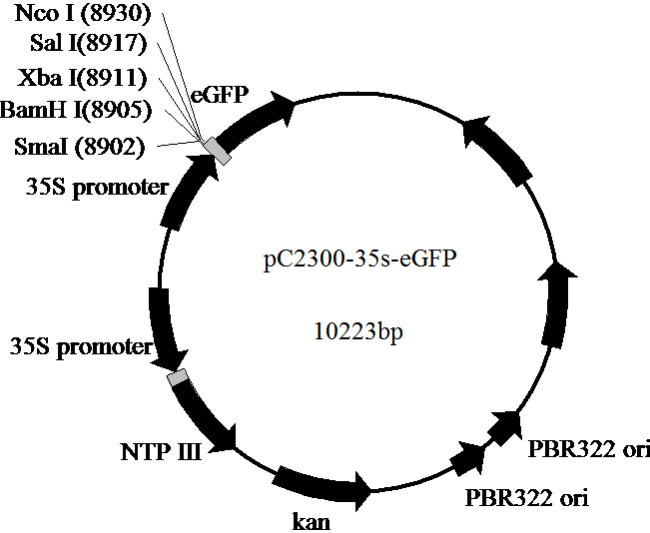

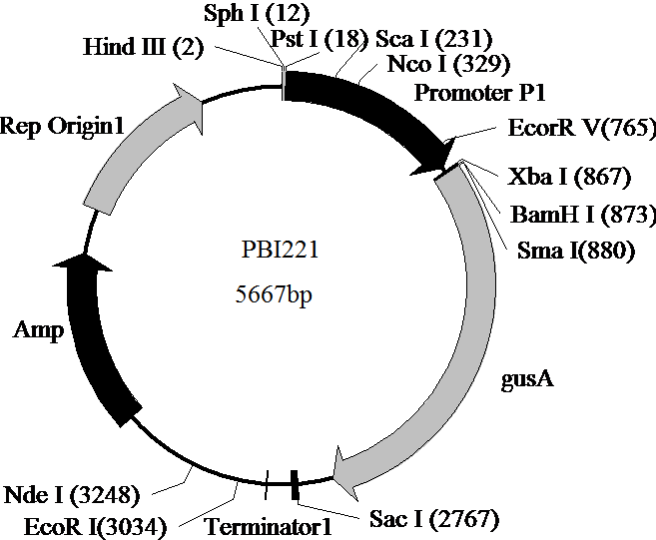


SUPPLEMENT 12 The name and JGI number of genes used to generate phylogenetic tree

| Name | JGI Number |
| --- | --- |
| Zm MYB-IF35 | GRMZM2G051256 |
| Zm MYB-IF25 | GRMZM2G051528 |
| Zm C1 | GRMZM2G005066 |
| P L | GRMZM2G701063 |
| P 1 | GRMZM2G084799 |
| P 2 | GRMZM2G057027 |
| Zm 42 | GRMZM2G419239 |
| Zm 31 | GRMZM2G050305 |
| Zm 148 | GRMZM2G097636 |
| Zm 111 | GRMZM2G104551 |
| Zm 1 | GRMZM5G833253 |
| At MYB12 | At2G47460 |
| At MYB11 | At3G62610 |
| At MYB58 | At1G16490 |
| At MYB111 | At5G49330 |
| Os C1 | AID46508.1 ( GenBank ) |
| At MYB85 | At4G22680 |
| At MYB75/PAP1 | At1G46650 |
| At MYB90/PAP2 | At1G66390 |
| At MYB4 | At5G26660 |
| At MYB32 | At4G34990 |
| At MYB63 | At1G79180 |
| Sl MYB12 | NP_001234401 |
| At MYB123/TT2 | At5G35550 |
| Pt MYB8 | ABD60280.1 ( GenBank ) |
